# Endogenous small-noncoding RNAs and their roles in chilling response and stress acclimation in Cassava

**DOI:** 10.1186/1471-2164-15-634

**Published:** 2014-07-29

**Authors:** Jing Xia, Changying Zeng, Zheng Chen, Kevin Zhang, Xin Chen, Yufei Zhou, Shun Song, Cheng Lu, Ruiju Yang, Zi Yang, Junfei Zhou, Hai Peng, Wenquan Wang, Ming Peng, Weixiong Zhang

**Affiliations:** Institute for Systems Biology, Jianghan University, Wuhan, Hubei 430056 China; The Institute of Tropical Bioscience and Biotechnology, Chinese Academy of Tropical Agricultural Sciences, Haikou, China; Department of Computer Science and Engineering, Washington University in St. Louis, One Brookings Drive, St. Louis, MO 63130 USA; Department of Genetics, Washington University School of Medicine, St. Louis, MO 63110 USA

**Keywords:** microRNA, tasiRNA, chilling acclimation, Cassava

## Abstract

**Background:**

Small noncoding RNA (sncRNA), including microRNAs (miRNAs) and endogenous small-interfering RNAs (endo-siRNAs) are key gene regulators in eukaryotes, playing critical roles in plant development and stress tolerance. Trans-acting siRNAs (ta-siRNAs), which are secondary siRNAs triggered by miRNAs, and siRNAs from natural antisense transcripts (nat-siRNAs) are two well-studied classes of endo-siRNAs.

**Results:**

In order to understand sncRNAs’ roles in plant chilling response and stress acclimation, we performed a comprehensive study of miRNAs and endo-siRNAs in Cassava (*Manihot esculenta*), a major source of food for the world populations in tropical regions. Combining Next-Generation sequencing and computational and experimental analyses, we profiled and characterized sncRNA species and mRNA genes from the plants that experienced severe and moderate chilling stresses, that underwent further severe chilling stress after chilling acclimation at moderate stress, and that grew under the normal condition. We also included castor bean (*Ricinus communis*) in our study to understand conservation of sncRNAs. In addition to known miRNAs, we identified 32 (22 and 10) novel miRNAs as well as 47 (26 and 21) putative secondary siRNA-yielding and 8 (7 and 1) nat-siRNA-yielding candidate loci in Cassava and castor bean, respectively. Among the expressed sncRNAs, 114 miRNAs, 12 ta-siRNAs and 2 nat-siRNAs showed significant expression changes under chilling stresses.

**Conclusion:**

Systematic and computational analysis of microRNAome and experimental validation collectively showed that miRNAs, ta-siRNAs, and possibly nat-siRNAs play important roles in chilling response and chilling acclimation in Cassava by regulating stress-related pathways, e.g. Auxin signal transduction. The conservation of these sncRNA might shed lights on the role of sncRNA-mediated pathways affected by chilling stress and stress acclimation in Euphorbiaceous plants.

**Electronic supplementary material:**

The online version of this article (doi:10.1186/1471-2164-15-634) contains supplementary material, which is available to authorized users.

## Background

Multicellular eukaryotes develop diverse small noncoding RNA (sncRNA) mechanisms for gene regulation at both DNA and RNA levels. In plants, most sncRNAs are generated by RNase III-type endonuclease DICERs or DICER-LIKE (DCL) proteins, and then incorporated into ARGONAUTE (AGO) proteins to exert their gene regulatory functions at the transcriptional level through DNA methylation and/or histone modification, and at the posttranscriptional level by mRNA cleavage, mRNA degradation or translational repression [1–5].

MicroRNAs (miRNAs) and endogenous small-interfering RNAs (endo-siRNAs) are two major classes of sncRNAs. miRNAs are typically processed from RNA polymerase II transcripts that fold into hairpin structures [6]. In plants, such hairpin-structured pre-miRNAs are then processed by DCL proteins in the nucleus to release ~22-nt double-stranded RNAs with ~2-nt 3′ overhangs, namely miRNA/miRNA* duplexes. The mature miRNAs are then loaded into the AGO-containing RNA-induced silencing complexes (RISC) in the cytoplasm to exert their regulatory effect by guiding the RISC to target transcripts through complete or partial complementary base pairing [7].

Endo-siRNAs have more diverse sources of origin. Endo-siRNAs arise from long double stranded RNAs (dsRNAs), which are formed from overlapped antisense transcripts or products of RNA-dependent RNA polymerases (RdRP) [8–11]. In plants, different classes of endo-siRNAs have been described based on their distinct characteristics, biogenesis pathways and functions [3, 12]. Trans-acting siRNAs (ta-siRNAs) and siRNAs from natural antisense transcripts (nat-siNATs) are two major classes of endo-siRNAs. Typically 21-nt in length and arranged in registers of 21-nt long phasing [7], ta-siRNAs are generated by phased Dicer processing of noncoding TAS genes or mRNA transcripts initiated by miRNAs [13–16].

Several genic regions have been annotated as ta-siRNA-yielding loci. In *Arabidopsis*, for example, a TAS3 gene has a pair of *miR390* binding sites, which define and produce a single RNA strand that is subsequently turned into a double stranded RNA by RDR6 polymerase. The double stranded RNA is subsequently cleaved by DCL4 to release a series of ~21-nt phased ta-siRNAs. Among these ta-siRNAs, one ta-siRNA species, named tasiARF, is broadly conserved and targets genes in the ARF family *in trans* [13]. Moreover, *Arabidopsis* has three TAS3 homologues (*AtTAS3a/b/c*) [16], rice carries three TAS3 loci [17], and *P. patens* has four (*PpTAS3a/b/c/d*) [13]. Besides TAS3, three other TAS genes (TAS1, TAS2 and TAS4) have been reported in *Arabidopsis*, while TAS3 is the only one broadly conserved, from rice and *Arabidopsis* to *P. patens* and Cassava [13]. ta-siRNAs may also arise from coding genes; a large number of genes encoding nucleotide binding site–leucine-rich repeat (NBS-LRR) plant innate immune receptors have been reported to give rise to ta-siRNAs, which were triggered by miR482 and miR2118 [16, 18, 19].

Endo-siRNAs can also be generated from cis-natural antisense transcripts (cis-NATs) [20–26]. These siRNAs, named as nat-siRNAs, can be induced by abiotic and biotic stresses [20, 27, 28] or can accumulate in specific developmental stages [23, 24]. The biogenesis of salt- and bacterium-induced nat-siRNAs in *Arabidopsis* requires DCL1 and/or DCL2, RDR6, and Pol IV [20, 28]. For example, the expression of *ARIADNE14* is de-repressed in *dcl1, hen1, hyl1, sde4, rdr2 and sgs3* mutants, suggesting that the nat-siRNAs from the cis-NAT pair *ARIADNE14 and KOKOPELLI* are dependent of DCL1, HEN1, HYL1, RDR2, SGS3 and PolIV [23].

Despite the broad existence of ta-siRNAs and nat-siRNAs in plant species, many of their features remain to be studied. It requires further effort to gain a comprehensive view of the genomic loci where ta-siRNAs and nat-siRNAs arise and to understand their regulatory roles in adaptation to dynamic environmental conditions. Further, little is known about the expression of miRNAs and endo-siRNAs in Euphorbiaceous plants. Here, we performed a comprehensive study of miRNAs, ta-siRNA and nat-siRNA in two agri-economic important Euphorbiaceous plants, Cassava (*Manihot esculenta*) and castor bean (*Ricinus communis*). Euphorbiaceae is one of the largest families in Angiosperms, consisting of more than 300 genera and about 7,500 species. Cassava is the most important crop in Africa and Southeast Asia and a primary source of food in most parts of the regions. Castor oil from castor bean has a medical use and is a critical raw material for many industrial products, such as lubricants and paints. Cassava and castor bean are capable of high photosynthesis and have complex traits for adapting to dynamic environments, making them ideal for studying small-RNA mediated gene regulation in stress response and stress acclimation. In the current study, we focus on identification and characterization of miRNAs, ta-siRNAs and nat-siRNAs in these two plants, particularly in Cassava, in response to chilling stress and in the process of chilling acclimation.

## Results

### Experiments to explore chilling response and stress acclimation

The study of miRNAs and endo-siRNAs in Cassava and castor bean was based on an integrative sncRNA and mRNA expression profiling experiment (Additional file 1: Figure S1 and Methods) [29]. Briefly, SC124, which is sensitive to chilling and is a widely planted Cassava cultivar in China, was subjected to three chilling stress treatments. The first was gradual *chilling acclimation* (CA) at a moderate stress where plants were subjected to a temperature decrease from 24°C to 14°C by -2°C/h and then grew for five days. In the second treatment of *chilling* stress after *chilling acclimation* (CCA), plants after the CA treatment were transferred further from 14°C to 4°C by -2°C/h and cultivated for another 5 days. In the third experiment, plants were subjected to *chilling shock* (CS) by a dramatic temperature drop from 24°C to 4°C with a gradient of -4°C/h. For comparison, plants grown under 24°C were taken as the *normal control* (NC). It is important to note that the three chilling treatments resulted in distinct phenotypes of elevated leaf proline content and/or malondialdehyde content [29].

### sncRNA and mRNA expression profiling by Next Generation sequencing

Four small-RNA libraries from the chilling-treated (i.e., CA, CCA and CS) and the normal (i.e., NC) plants of SC124 were prepared and sequenced separately using Illumina Genome Analyzer IIx (GAIIx) (see Methods, sequencing data in NCBI/GEO, accession # GSE52178). These libraries contributed to more than 25.6 million raw small-RNA reads total, among which 23,468,606 (>91% of the total) were adapter-trimmed, high-quality reads (qualified reads, Additional file 2: Table S1A). Among the qualified reads, 53.40% and 73.18% could map to the Cassava reference genome (http://www.phytozome.net) allowing zero and one mismatch (Additional file 2: Tables S1B and S1C), respectively, indicating a high sequencing quality despite that SC124 is different cultivar from the reference genome AM560. The qualified and genome mapped reads had lengths peaked at 21-nt and 24-nt, and carried twice more U’s and A’s than G’s and C’s as the first nucleotides (Additional file 1: Figures S2A and S2B). In comparison, the reads from miRNAs were dominantly 21-nt or 22-nt and carried preferentially U’s at the first nucleotides (Additional file 1: Figures S2C and S2D).

To appreciate the potential regulatory effects of sncRNAs, four mRNA libraries, which were prepared using the plants from the same three chilling treatments and the normal condition, were sequenced individually using the Illumina RNA-seq protocol (see Methods, sequencing data in NCBI/GEO, accession # GSE52178). Briefly, from more than 80% genome-mapped reads of more than 35.3 million raw reads, 12,689 (37.16% of the 34,151 annotated Cassava mRNA genes), 16,023 (46.92%), 15,144 (44.34%) and 17,026 (49.85%) mRNA genes were expressed under the NC, CA, CCA, and CS conditions, respectively (Additional file 2: Table S2, see Methods). Among the expressed genes were 2855, 1082 and 3297 differentially expressed genes in AC, CCA and CS in reference to NC (Additional file 2: Table S2). These differentially expressed genes were further analyzed, in addition to sncRNAs, in the study.

sncRNA species expressed in CCA treated and the normal castor bean plants were profiled following the same sequencing protocol as used for Cassava. The sequencing data, which have similar percentage of qualified reads and reads mapped to the reference genome and similar distributions of length and first nucleotide bias as those of Cassava, with one exception that miRNAs were dominantly 21-nt long in castor bean (Additional file 1: Figure S3 and Additional file 2: Table S3).

### Novel miRNAs in Euphorbia and their conservation

#### Novel miRNAs

Utilizing the sequencing data and a set of stringent criteria (see Methods), we identified 22 and 10 novel miRNAs in Cassava and castor bean, respectively (Table 1A and B). Alignments of novel miRNA sequencing reads to the respective genomes are given in Additional files 3 and 4. If a newly detected miRNA has been reported in other plants, e.g., miR3627, the same family name was used; otherwise, a temporary new family name, e.g., novel-6, was introduced. The same temporary family names were used for Cassava and castor bean to indicate their conservation across the two species. For example, mes-novel-40 in Cassava (Table 1A) and rco-novel-40 in castor bean (Table 1B) belong to the same miRNA family. Among these novel miRNAs, four novel miRNAs in Cassava, novel-20, novel-52, novel-54, and novel-55, were highly represented as detected by sequencing reads (Table 1A). The sequence reads for these four miRNAs counted for 6% of the total reads from Cassava miRNAs.

**Table 1:** **Novel miRNAs in Cassava and castor bean identified from small-RNA profiling**

No.	Mature_sequence	#. Reads	WGS_ID	Start	End	Strd.	Genomic region	Validation	Name
**(A) Twenty-two novel miRNAs were identified and experimental validated in Cassava**									
**1**	UGGACGCCAUUUUGACAGAUG	248	scaffold00847	1153336	1153495	+	Intergenic	N	novel-3
**2**	CAAAUUAUAAUGGCAUUUUGA	11	scaffold02022	100612	100777	+	Intergenic	nd	novel-10
**3**	UGGGUAAGUGGGGAAGAUAAC	90	scaffold02264	510281	510416	+	Intergenic	N	novel-11
**4**	AAAUGGGACUCAUCAUAUGGUGGG	50	scaffold02658	994069	994318	+	intron.2_Cassava 4.1_019698m.g	Y	novel-14
**5**	UGGCCUAGAGUAGUGACCUCC	346	scaffold02936	84627	84693	-	Intergenic	Y	novel-16
**6**	AGAUGGGUGGCUCGGGAAGAAG	21209	scaffold03604	533307	533466	-	Intergenic	Y	novel-20
**7**	UUAUUUGAUCAAGGGAAAUUC	154	scaffold03802	905079	905143	+	3'UTR.1_Cassava 4.1_011822m.g	N	novel-21
**8**	UUUGGGGUAAAUUUGGACCAAA	46	scaffold05335	14983	15180	-	intron.4_Cassava 4.1_025731m.g	N	novel-24
**9**	UGGCCUUUUGAGUUUGAGAAGACA	100	scaffold05694	371116	371230	-	intron.6_Cassava 4.1_022022m.g	N	novel-27
**10**	UUGGAAGAGCUUACUUUAAAU	495	scaffold05875	2354583	2354832	-	Intergenic	N	novel-28
**11**	UCUGAAUCCCUGACGAAGCCU	243	scaffold05884	79762	79829	+	Intergenic	Y	novel-29
**12**	UUUAUAUCAUGCAUAAUUAAG	82	scaffold05890	126	236	-	Intergenic	N	novel-30
**13**	UGUCGCUGGAGAAAUGGCACUA	80	scaffold07240	11314	11420	-	Intergenic	nd	novel-38
**14**	UUUUAAUGAUAGUAUAGGGGU	12	scaffold07290	137355	137549	+	Intergenic	nd	novel-39
**15**	UGGGUGGGUGAGUGGAUAAGA	172	scaffold07996	160667	160818	-	Intergenic	Y	novel-40
**16**	UCCAGGCAAGGAAAGCUUUUC	28	scaffold08542	27109	27229	-	Intergenic	N	novel-44
**17**	UUGAGGGCUGUUUCCAGAAGC	207	scaffold10241	178034	178193	+	Intergenic	Y	novel-50
**18**	UCUAUAUGGUCUGCGGUUACC	219	scaffold12301	237927	238086	+	Intergenic	Y	novel-51-5p
**18**	UGACCGCAGACCAUAUAGAAC	446	scaffold12301	237927	238086	+	Intergenic	Y	novel-51
**19**	GGAAUGGGCGGUUUGGGAAAA	29917	scaffold04043	391309	391458	-	Intergenic	Y	novel-52
**19**	UUCCCAAUGUCGCCCAUUCCGA	890	scaffold04043	391309	391458	-	Intergenic	Y	novel-52-3p
**20**	AAAAGGAAGAUGGAGGGCAUGA	126	scaffold03264	387326	387485	-	Intergenic	nd	novel-53
**21**	ACUCUCCCUAAAGGCUUCAAC	3851	scaffold03581	762705	762813	-	Intergenic	Y	novel-54
**22**	UAUGGGGGGAUUGGGCAAAAU	38040	scaffold03604	533082	533222	-	intergenic	Y	novel-55-5p
**22**	UUCCCAAGACCUCCCAUACCAG	654	scaffold03604	533082	533222	-	intergenic	Y	novel-55-3p
**No.**	**Mature_sequence**	**#. Read**	**WGS_ID**	**Start**	**End**	**Strd.**	**Genomic region**		**Name**
**(B) Ten novel miRNAs in castor bean**									
**1**	UGGGUGAGUGGAGAAGAUAAC	14	30128	2013705	2013875	-	intergenic		novel-40
**2**	GGAAUGGGCGGUUUGGGAAAG	3622	29586	144967	145126	-	intergenic		novel-52
**3**	ACUCUCUCUGAAGGCUUCAAA	4755	29742	519324	519418	-	intergenic		novel-54
**4**	UAUGGGGGGAUCGGGCAAUAUU	577	29660	174480	174647	+	intergenic		novel-55-5p
**4**	UCUUCCCGAGACCUCCCAUACC	281	29660	174480	174647	+	intergenic		novel-55-3p
**5**	UCUUAUAGCAAUCAGGGGACUUG	296	29877	66504	66639	+	intergenic		novel-63
**6**	UAGCAAAAGAUAGAACCGGAG	225	29904	1112560	1112809	+	intergenic		novel-64
**7**	UCUGAUAGCAAAAGAUAGAAC	528	29904	1112560	1112809	-	intergenic		novel-64as
**8**	CGAGUCAUCUGACAGAAGUAG	5102	29912	1877312	1877561	+	intergenic		novel-65
**9**	UGACGUGGCAUGAACUUCGGCA	756	30074	662171	662379	-	intron.6_30074.t000092		novel-66
**10**	UCCUCUGUCACAAAUGGCUUCCAG	182	29912	1868939	1869188	+	intergenic		novel-67

Included in (A) and (B) are the number of qualified reads from all small-RNA libraries (#. reads), the genomic scaffold ID (WGS_ID), the start and end positions of the hairpins, strand (strd.) and genomic region where a miRNA resides. Included also in (A) and (B) are mature miRNA sequences, named as miR*n*-5p and miR*n*-3p, if both sequences had a substantial number of reads. The “Y” or “N” indicates whether the novel miRNA has been detected or not, respectively, in at least one of four chilling treatment samples, and novel miRNAs not selected for validation are marked with “nd”.

#### Re-annotation of known miRNAs

We further analyzed the Cassava and castor bean miRNAs identified in our previous study [30] (miRBase version 20), which were detected by analyzing our deep sequencing data. Furthermore, 17 recently reported candidate miRNAs not in miRBase [31–33], which were named as reported-*k* to distinguish them from the ones in miRBase, were also presented in our sequencing data (Additional file 2: Table S4). Among them, a newly reported candidate, reported-31 [31], counted for 138,426 of the total reads and ranked the 4-th most abundant among all miRNA families that were detected; it was only less abundant than miR156i/j/k, miR166 (all eight members), and miR167d/e/f (Additional file 2: Table S4). Besides, 163 individual miRNAs, belonging to 33 families in Cassava were listed in Additional file 2: Table S4. The miR156, miR171, miR166, and miR169 families constitute the largest miRNA families in Cassava (Table 2), with 11, 11, 11, and 29 members, respectively. All these miRNAs in Cassava and castor bean were re-annotated according to miRNA identification criteria (Additional file 2: Tables S4 and S5). Alignments of miRNA sequencing reads to the respective genomes are given in Additional files 5 and 6.

**Table 2:** **Conservation of Euphorbiaceous miRNA families across 9 plant species –** ***M. esculenta*** **(mes),** ***R. communis*** **(rco),** ***P. trichocarpa*** **(ptc),** ***M. truncatula*** **(mtr),** ***G. max*** **(gma),** ***A. thaliana*** **(ath),** ***V. vinifera*** **(vvi),** ***O. sativa*** **(osa) and** ***P. patens*** **(ppt)**

miR	mes	rco	ptc	mtr	gma	ath	vvi	osa	ppt
**156**	**11**	**8**	**11**	**9**	**7**	**8**	**9**	**12**	**3**
**159**	**4**	**1**	**6**	**1**	**4**	**3**	**3**	**6**	
**160**	**8**	**3**	**8**	**5**	**1**	**3**	**5**	**6**	**9**
**162**	**2**	**1**	**3**	**1**	**1**	**2**	**1**	**2**	
**164**	**4**	**4**	**6**	**4**	**1**	**3**	**4**	**6**	
**166**	**11**	**5**	**17**	**8**	**2**	**7**	**8**	**14**	**13**
**167**	**9**	**3**	**8**	**1**	**7**	**4**	**5**	**10**	
**168**	**1**	**1**	**2**	**1**	**1**	**2**	**1**	**2**	
**169**	**29**	**3**	**32**	**17**	**5**	**14**	**25**	**17**	
**171**	**11**	**7**	**14**	**7**	**3**	**3**	**9**	**9**	**2**
**172**	**6**	**1**	**9**	**1**	**6**	**5**	**4**	**4**	
**319**	**8**	**4**	**9**	**2**	**3**	**3**	**5**	**2**	**5**
**390**	**3**	**2**	**4**	**1**	**2**	**2**	**1**	**1**	**3**
**393**	**4**	**1**	**4**	**2**	**1**	**2**	**2**	**2**	
**394**	**3**	**2**	**2**		**2**	**2**	**3**	**1**	
**395**	**5**	**5**	**10**	**18**	**3**	**6**	**14**	**25**	**1**
**396**	**6**	**1**	**7**	**2**	**5**	**2**	**4**	**9**	
**397**	**2**	**1**	**3**		**2**	**2**	**1**	**2**	
**398**	**1**	**2**	**3**	**3**	**2**	**3**	**3**	**2**	
**399**	**7**	**6**	**12**	**17**		**6**	**9**	**11**	
**403**	**2**	**2**	**3**		**2**	**1**	**6**		
**408**	**2**	**1**	**1**	**1**	**4**	**1**	**1**	**1**	**2**
**477**	**9**	**1**	**2**				**1**		**8**
**482**	**1**		**4**	**1**	**5**		**1**		
**530**	**2**	**1**	**2**		**2**			**1**	
**535**	**3**	**1**					**3**	**1**	**4**
**827**	**1**	**1**	**1**			**1**		**3**	
**828**	**2**	**2**	**2**		**2**	**1**	**2**		
**1446**	**1**	**1**	**5**						
**2111**	**2**	**1**		**19**	**1**	**2**	**1**		
**2275**	**1**							**1**	
**2950**	**1**						**1**		
**3627**	**1**	**2**	**2**				**1**		

Listed in the tables are the numbers of individual members of miRNA families.

#### Experimental validation and analysis of miRNAs

**Figure 1:**
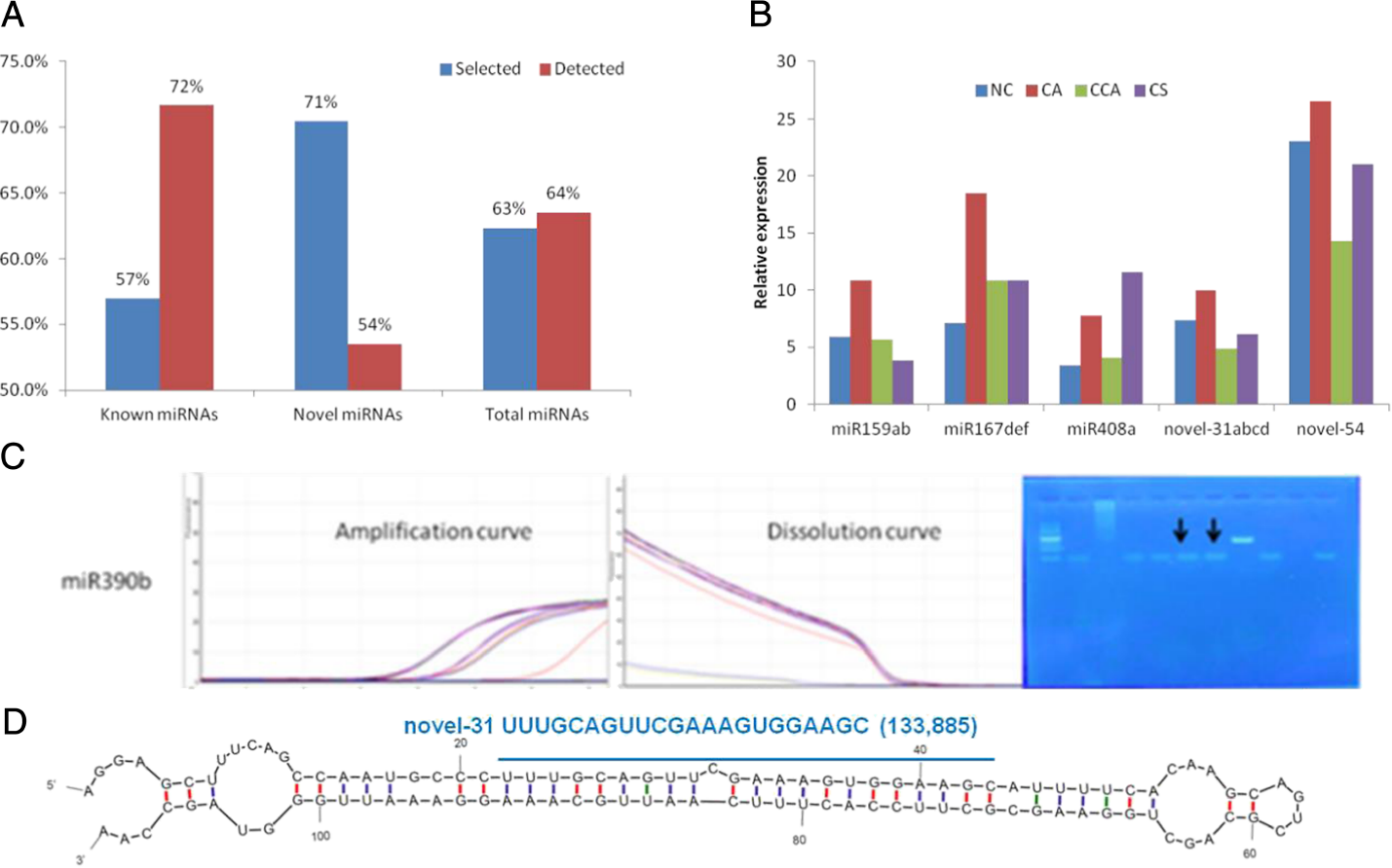
**Experiment validation of sncRNAs in Cassava cultivar SC124. (A)** Expression of miRNAs confirmed by qRT-PCR validation, where “selected” is the percent of miRNAs selected for qRT-PCR assays from all expressed miRNAs based on sequencing data, and “Detected” is the percent of the selected miRNAs that can be detected by qRT-PCR in at least one of the four conditions. **(B)** Examples of validated known and novel miRNAs expressed in three chilling treatments and the normal condition. **(C)** Amplification curve, dissolution curve, and endpoint gel image of amplicon products of miRNA novel-55. Solid arrows indicate the target band in gel. **(D)** The hairpin structure of novel-55 with annotated miRNA-5p highlighted in blue. The number in parenthesis represents the number of reads in the sequencing data.

We selected 95 known, newly reported and novel miRNAs to validate and assess miRNA’s expression under four conditions in SC124 with qualitative real-time RT-PCR (qRT-PCR) methods. As a result, 61 out of 95 miRNAs were detected in at least one of the four conditions, respectively (Figure 1A and Additional file 2: Table S6). Figure 1B shows examples of known and novel miRNAs by qRT-PCR, and Figure 1C displays the detailed experimental results on novel miRNA novel-55 whose hairpin structure is shown in Figure 1D.

For known miRNAs, the detected miRNAs have a large number of reads (an average of 26,838 reads), but miRNAs that failed to be detected have smaller number of reads (an average of 2,869 reads). For example, two miRNA families (miR159 and miR167), which had more than 7 K reads, can also be detected in qRT-PCR validation (Figure 1B, Additional file 2: Tables S4 and S6). Similar to known miRNAs, the detected novel miRNAs had large numbers of reads (with an average of 28,501 reads), but undetected miRNAs typically had small numbers of reads (with an average of 137 reads). Mature miRNAs may arise to an abundant level from both arms. For example, four miRNAs (novel-51, novel-52, novel-54 and novel-55), each of which had more than 200 reads, had their mature miRNAs detected by qRT-PCR (Additional file 2: Table S6). Taken together, miRNAs with low numbers of sequencing reads were likely not to be detected by qRT-PCR, reflecting that deep sequencing was more sensitive to low abundant transcripts.

#### Conservation of miRNAs

The conservation of all known, newly identified and novel miRNAs in Cassava and castor bean was examined in reference to seven diverse plant species, i.e., Populus, Medicago, soy bean, *Arabidopsis*, grapes, rice and moss (*Physcomitrella patens*) (Table 2). Eight known miRNA families – miR156, miR160, miR166, miR171, miR319, miR390, miR395, and miR408 – were evolutionally conserved across the 9 species (Table 2). Another 10 annotated miRNA families – miR159, miR162, miR164, miR167, miR168, miR169, miR172, miR393, miR396, and miR398 – were also conserved in at least eight species (Table 2). The highly expressed report-31 had no homologs in castor bean and the other seven plants. The conservation of these miRNAs is broad, ranging from the angiosperm lineages to vascular plants, suggesting their essential functions in plants [15].

Remarkably, most novel miRNA families identified in the current study were Euphorbia specific. In these miRNA families, five (novel-14, novel-40, novel-52, novel-54, and novel-55) exist in both Cassava and castor bean, while 18 and 6 novel miRNAs were Cassava and castor bean specific, respectively (Table 1). Among the highly expressed novel miRNAs in Cassava (Table 1A), three (novel-52, novel-54, and novel-55) had homologs in castor bean, though they did not appear in other plants beyond Euphorbia. The existence of lineage/species specific miRNAs in Cassava and castor bean indicated some specific roles that these miRNAs may play in stress response in Euphorbiaceous plants.

### MicroRNA-triggered secondary siRNAs

A genome-wide search of small RNAs resembling secondary siRNAs (see Methods) resulted in 26 and 21 genomic loci enriched with 21-nt long in length in the small-RNA libraries of Cassava and castor bean, respectively (Table 3, Additional file 2: Tables S7 and S8). These genomic loci were targeted by 17 miRNA families in Cassava (Additional file 2: Table S7) and 12 miRNA families in castor bean (Additional file 2: Table S8). The majority (85%) of these siRNA-generating genomic loci in Cassava correspond to protein-coding genes (Table 3). These genes fell into several classes. The first was the set of three loci corresponding to the TAS3 genes, known hosts of ta-siRNAs, despite that only one TAS3 locus has been annotated in the current Cassava reference genome [13]. The second and biggest class consisted of 10 NB-ARC domain-containing disease resistance genes, which are receptors sensing intracellular perturbations [34]. The third class contained 2 auxin response factors and 2 auxin signaling F-box genes.

**Table 3:** **Distribution of genes that are targeted by miRNAs and subsequently produce candidate secondary siRNAs in (A) Cassava and (B) Castor bean, and their function categories**

#. Genes	Transcripts yielding 21-nt enriched siRNAs
**A. Cassava**	
2	ATPase E1-E2 type family protein/haloacid dehalogenase-like hydrolase family protein
2	auxin response factor 8
2	auxin signaling F-box 3
1	dicer-like 1
2	F-box/RNI-like superfamily protein
1	GRAS family transcription factor
10	NB-ARC domain-containing disease resistance protein
1	phosphate transporter 1;3
1	scarecrow-like 3
4	TAS3 and unannotated loci
**B. Castor bean**	
3	auxin response factor
1	auxin signaling F-box
1	basic helix-loop-helix (bHLH) DNA-binding superfamily protein
1	Cupredoxin superfamily protein
1	disease resistance protein (TIR-NBS-LRR class), putative
1	Galactose oxidase/kelch repeat superfamily protein
2	GRAS family transcription factor
1	Integrase-type DNA-binding superfamily protein
1	Leucine-rich receptor-like protein kinase family protein
1	Malectin/receptor-like protein kinase family protein
1	SET domain protein
3	squamosa promoter binding protein-like
1	TCP family transcription factor
3	TAS3

Many of these siRNA-generating loci and their targeting miRNA candidates were conserved in castor bean (Table 3). As expected, the three TAS3 loci and the targeting miRNA (i.e., miR390), 3 auxin response factors, and auxin signaling F-box genes were targeted by miR167 and miR393 and two GRAS family transcription factors were targets of miR171. Such a strong conservation suggested some common regulatory functions of those siRNA-yielding loci in Euphorbiaceous plants.

#### ta-siRNAs triggered by miR390 and Novel TAS3 genes

A genome-wide homologue search of TAS genes in Euphorbia concluded that TAS1 and TAS2 genes did not exist in the Cassava and castor bean genomes. This is consistent with the fact that miR173, which triggers ta-siRNAs in homologies of TAS1 and TAS2 in *Arabidopsis*, is not conserved in Euphorbia [15]. TAS4 did not seem to appear in Cassava or castor bean either, although miR828, the initiator of TAS4-siRNAs in *Arabidopsis*, exists in Cassava and castor bean [15]. One TAS3 gene is currently annotated in the Cassava reference genome (gene ID: 019138 m, [13]). This is in sharp contrast with three TAS3 genes in *Arabidopsis* (*AtTAS3a/b/c*) [16] and four TAS3 genes in *P. patens* (*PpTAS3a/b/c/d*) [13].

**Figure 2:**
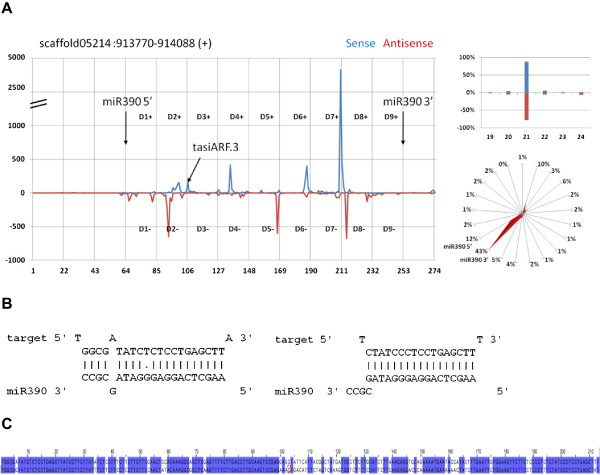
**miR390-triggered siRNAs from** ***TAS3c*** **in Cassava. (A)** Distribution of small-RNA reads. Plotted are the 5′ ends of the reads at each position along the sense (in blue) and antisense (in red) strands within the *TAS3c* region. The two arrows marked with miR390 indicate the cleavage sites of miR390 on *TAS3c*.The distribution of the lengths of these small RNAs is shown in the right-top figure. The radial graph in the right-bottom shows the percent of small RNAs with 5′ ends aligned to one of the 21-phasing registers. The registers to which 5′ and 3′ miR390 cleavage sites are aligned are indicated by miR390 5′ and miR390 3′. **(B)** The two target sites (top) and base pairing of miR390 (bottom) on *TAS3c* in Cassava. Sense (in blue) and antisense reads (in red) alignments are plotted near the two target sites. The arrows indicate the putative cleavage sites. Phased siRNA reads duplex with ~2 nt 3′ overhang can be observed on the sense (blue) and antisense (red) strands. The yellow colored are the miR390 targets. **(C)** Conservation of TAS3c between Cassava and Castor bean. The regions marked blue are conserved with identical bases in the two species.

We identified two novel TAS3 loci in both Cassava and castor bean genomes by searching for a pair of miR-390 target sites (Figures 2, Additional file 1: Figures S4, S5 and S6). The currently annotated TAS3 gene was thus named as *TAS3a* and the two new TAS3 genes as *TAS3b* (Additional file 1: Figures S4 and S5) and *TAS3c* (Figure 2 and Additional file 1: Figure S6). Importantly, a substantial number of sequencing reads, arranged in phasing, appeared in the regions defined by the two miR390 target sites (Figure 2A, Additional file 1: Figures S4A, S5A and S6A). Following the standard nomenclature [35], the register of phased siRNAs was named as D1, D2, and so on, starting from the 5′-end miRNA target site, and the orientation was indicated by suffix “+” for the positive (or the original transcript) strand, or “-” for the negative (or the RdRP synthesized) strand.

The two binding sites of miR390 on TAS3c gene have a highly complementary sequence match (Figure 2B, left panel). Moreover, nearly perfect matches around the 10-nt from the 5′-end of miR390 binding sites suggested that both binding sites were cleaved, which were further supported by the phased sequencing reads starting at the position of the cleavage sites of both 5′- and 3′-end target sites (Figure 2B, right panel). These two-cleavage sites have been reported for TAS3 genes in *P. patens* [13] but different from that of *Arabidopsis* TAS3a where only the 3′-end target site is cleaved [7]. Similar cleavage sites of miR390 on the TAS3c gene appeared in castor bean (Additional file 1: Figures S6D and S6E).

Interestingly, the regions between the two pairs of miR390 target sites on *TAS3c* in the two Euphorbiaceous species had the same length of 190-nt and shared a high sequence identity (Figure 2C). The regions around the two target sites had almost identical sequences, suggesting a strong conservation on and near the miR390 target sites. Importantly, siRNA from D4+ register (Figure 2A) was highly conserved with tasiARF, a well-studied ta-siRNA targeting an ARF gene in *Arabidopsis* (*ASRP2141*) [7]. It is noteworthy that TAS3a and TAS3b also host tasiARF-like siRNAs in Euphorbia. However, sequence alignment revealed that there existed three distinct tasiARF-like siRNA species encoded by TAS3a/b/c; a slight sequence difference near the 3′-ends existed in the three tasiARFs (Additional file 1: Figure S7). Finally, the three TAS3 genes encoded different numbers of tasiARF. TAS3a and TAS3b maintained two tasiARFs adjacent to each other (e.g., tasiARF.1 and tasiARF.2 in Additional file 1: Figures S4A and S5A), while TAS3c contained a single tasiARF (Figure 2A).

A total of 8,479 reads from the small-RNA libraries of Cassava were mapped to the *TAS3c* region, among which 85.1% were 21-nt long. Among these small RNAs, 5,884 (66.4% of the total of 8,479) were arranged in phase with reference to the putative cleavage site at either the 5′ or 3′ target site of miR390. Interestingly, 20.5% of 21-nt small RNAs have the first nucleotides at the positions shifted by ~10- or 11-nt from the majority phased small RNAs. These shifted siRNAs followed the phasing register set by siRNA D8-, indicating that siRNAs arising from D8- could target and cleave the original transcripts, setting a secondary phasing register different from the register set by miR390. The most prominent example of such shifted siRNAs was located at the middle of D5+ and D3- (Figure 2A). Similarly, 2,195 sequencing reads, 81% of which were 21-nt long, were derived from TAS3c in castor bean. Secondary, shifted siRNAs (21% of the total siRNAs from TAS3c) were also observed (Additional file 1: Figure S6C), similar to that in Cassava TAS3c, exemplified by the peaks in the middle of D6+, D6- and D5-. Taken together, TAS3c in Cassava and castor bean produced ample ta-siRNAs, majority of which were arranged in 21-nt phasing, while a small amount of which had a register shifted half way from the main register.

#### Candidate secondary siRNAs triggered by miRNAs

A total of 77 and 86 NBS-LRR genes are annotated in the current Cassava and castor bean genomes, respectively [36]. However, only one castor bean NBS-LRR genes was predicted to be targeted by miR396 to produce enriched siRNAs of 21-nt long (Table 3). This is in contrast to the results in Medicago and tomatoes, where a large number of NBS-LRR genes have been reported to be targeted by one or two miRNA initiators, such as miR482 [18, 19]. We were able to identify one locus encoding miR482 in Cassava genome (Additional files 5). However, miR482 in Cassava was rarely sequenced in the current data sets (Additional file 5), indicating that miR482 might not be expressed in the tissues we examined. Alternatively, it is plausible that miR482 did not regulate NBS-LRR genes in Euphorbiaceous species, which is consistent with the prediction that few NBS-LRR genes were targeted by miR482 in Cassava or castor bean, respectively.

ta-siRNAs at TAS3 loci were found to be arranged in phasing. Nevertheless, many other siRNAs were not arranged in a precise 21-nt phasing pattern (Additional file 2: Table S7). For example, an unannotated genomic locus associated with report-31 produced 14,369 siRNA reads in total, 84% of which were 21-nt long (Additional file 1: Figures S8A and S8B), but interestingly, the siRNAs were not arranged in phasing (Additional file 1: Figures S8C), different from the phasing pattern of siRNAs from the TAS3 loci. Non-phasing ta-siRNAs have also been observed in *Arabidopsis*, where RDR6-dependent siRNAs derived from miRNA target loci (e.g. miR168 at AGO1, miR472 at several CC-NBS-LRR genes) were found not to be arranged in a phasing pattern either [16]. Therefore, we reported both phased and non-phased siRNA candidates from miRNA-targeted genes.

#### Experimental validation of secondary siRNAs and their targets

We experimentally validated three ta-siRNAs including tasiARF1/2 from TAS3a/b genes and the highly sequenced D8+ siRNA from the newly identified TAS3c gene (Additional file 1: Figure S9). The qRT-PCR result showed consistent amplification and sharp dissolution curves as well as clear bands of three siRNAs, supporting the genuine presence of the three ta-siRNAs (Additional file 1: Figure S9).

**Figure 3:**
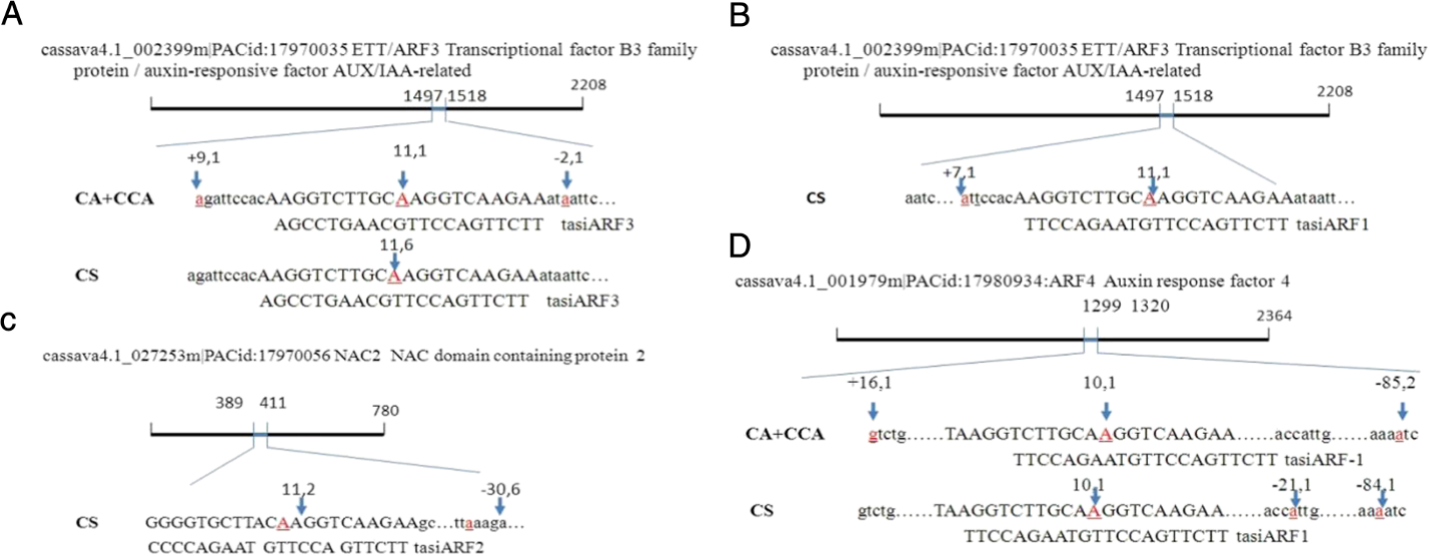
**Cleavage sites of ta-siRNAs detected by 5′-RACE in chilling-treated Cassava plants.** Shown here are the cleavage sites of **(A)** tasiARF3 on ARF3 (002399 m), **(B)** tasiARF1 on ARF3 (002399 m), **(C)** tasiARF2 on NAC2 (027253 m), and **(D)** tasiARF1 on ARF4 (001979 m). In each panel, the name of a target, its annotation and the ta-siRNA-binding region are shown on the top and the reversed ta-siRNA sequence on the bottom with conditions shown to the left (CA, CCA and/or CS). An arrow indicates a cleavage on the target gene. The first number atop of an arrow refers to the cleavage site within the ta-siRNA-binding region from the 5′ end of the small RNA, and the following number is the number of clones (out of 10) detected by cloning of amplified the specific PCR product. A number with prefix “+” or “-” refers to, respectively, the cleavage site located upstream or downstream of the base-pairing region on the target gene. The number on a solid line indicates the distance between the cleavage site and the boundary of ta-siRNA-binding region.

Further, to investigate the effect of sncRNAs on target genes, we used 5′-RACE to assess the cleavage activities of miRNAs and secondary siRNAs of TAS3 on their targets. The miRNAs that we tested could indeed cleave their targets, as shown by the cleavage sites of miR167 on ARF8 and that of miR393 on ABF3 (Table 4). The two putative cleavage sites of miR390 on TAS3c were cleaved (Table 4), in agreement with the reads from the RNA-seq data (Figure 2B). Moreover, ta-siRNAs of TAS3 were also able to cleave their target genes. The subsequent secondary ta-siRNAs – tasiARF1, tasiARF2, and tasiARF3 – cleaved their respective targets; specifically, tasiARF1 cleaved both ARF3 and ARF4, tasiARF2 cleaved NAC2 and tasiARF3 cleaved ARF3 (Table 4 and Figure 3). Interestingly, tasiARF1 and tasiARF3 degraded the same target (AFR3, 002399 m) at the same cleavage site (the 11-th nucleotide from the 5′ end of the small-RNA binding site) with different cleavage efficiency (i.e., approximately by a 6 to 1 ratio) under the CS condition (Table 4, Figure 3A and 3B). Moreover, one putative secondary siRNA derived from report-31 initiated transcript, i.e., siReport-31, was able to cleave its target gene, CUL4. Note that some cleavage sites detected by 5′-RACE were separated by consecutive segments of 21-nt long, e.g., between the cleavages sites at 21-nt and 84-nt downstream from the tasiARF1 cleavage sites on ARF4 (data not shown).

**Table 4:** **miRNAs and siRNAs cleavage sites detected by 5′RACE**

Genes	Condition	Basepair	Target ID		Annotation
miR390-TAS3c binding site #1	CA + CCA	1(1)	scaffold05214	TAS3c	Non-coding gene, the inter-space between NO.1 and NO.2 basepair match is 168 bp.
	CS	11(2), 1(6), 0(1)			
miR390-TAS3c binding site #2	CA + CCA	5(1)			
	CS	5(1)			
miR167-ARF8	CA + CCA	9(1), 7(1)	001923 m	ARF8	auxin response factor 8
miR393-ABF3	NC	10(1), 17(1)	004520 m	AFB3	auxin signaling F-box 3
	CA + CCA	11(8)			
	CS	11(7)			
tasiAFR3-ARF3	CA + CCA	11(1)	002399 m	ETT/ARF3	Transcriptional factor B3 family protein/auxin-responsive factor AUX/IAA-related
	CS	11(6)			
tasiARF1-ARF3	CS	11(1)	002399 m	ETT/ARF3	
tasiARF1-ARF4	CA + CCA	10(1)	001979 m	ARF4	auxin response factor 4
	CS	10(1)			
tasiARF2-NAC2	CS	11(2)	027253 m	NAC2	NAC domain containing protein 2
siReport31-CUL4	CA + CCA	18(1)	001768 m	CUL4	cullin4

Each target site was detected by ten clones. “Basepair” column lists the specific cleavage site from the 5′ end of a miRNA or siRNA and the number of clones (in parenthesis) that were detected to have cleavage products.

### Candidate siRNAs originated from natural antisense transcripts

Following our method for identifying cis-NATs and using the threshold of 25-nt overlapping [26], 135 and 63 pairs of cis-NATs were identified, respectively, in the Cassava and castor bean reference genomes (Additional file 2: Tables S9 and S10). Among the 135 cis-NATs in Cassava, 48 (36%), 15 (11%) and 72 (53%) pairs were arranged in the convergent (3′-3′ overlap), divergent (5′-5′ overlap) and enclosed orientations, respectively (Additional file 2: Tables S9). Note that the percentage of enclosed cis-NATs was greater than that in *Arabidopsis* [25, 26]. In castor bean, the same number of 23 cis-NAT pairs appeared in the enclosed and divergent categories and 17 were convergent (Additional file 2: Table S10).

**Figure 4:**
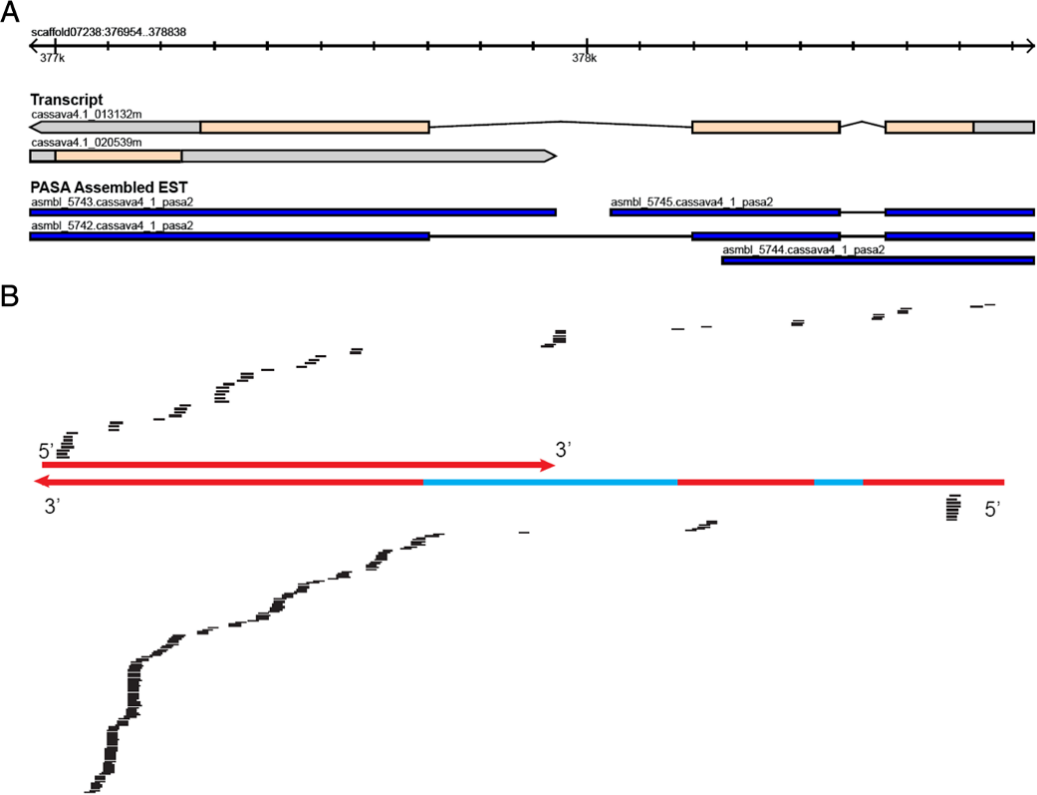
**A cis-NAT candidate locus yields 21-nt enriched siRNAs. (A)** cis-NAT pair of no apical meristem gene (NAM, 013132 m) and unannotated gene (020539 m) in Cassava. The EST track displays the EST sequences from the Phytozome database [36]. **(B)** Small RNAs from the positive and negative strands are displayed above and below the NAT pairs, respectively. The red and blue regions on the gene model represent exons and introns, respectively.

Seven and 1 cis-NAT in Cassava and castor bean, respectively, were detected to yield nat-siRNAs, each of which gave rise to at least 10 reads in the overlapping region (Table 5). One example of cis-nat-siRNAs was from the cis-NAT pair of two genes, 013132 m and 020539 m on scaffold07238 (cis-NAT-1 in Table 5), arranged in a convergent fashion (Figure 4); a total of 271 reads aligned to the overlap region of the two genes. Approximately 80% of the mappable reads appeared in the cis-NAT overlapping region, showing that cis-nat-siRNAs were predominantly derived from the overlapping region. Transcription of the two NAT genes were confirmed by Expressed Sequence Tags (ESTs; accession number asmbl_5742 and _5743) and directly supported by our RNA-seq data of the Cassava transcriptome profiling under the four conditions (Additional file 2: Table S11).

**Table 5:** **cis-NAT pairs that generate siRNAs in Cassava and castor bean**

						+ Strand		- Strand
ID	Scaffold	Type	Start	End	# Reads in OL ^a^	21 nt% ^b^	# Reads in OL ^a^	21 nt% ^b^
***Cassava***								
cis-NAT-1	scaffold07238	3′-3′	376957	377910	40	85.0%	175	66.3%
cis-NAT-2	scaffold11378	3′-3′	285315	286689	53	57.2%	10	60.0%
cis-NAT-3	scaffold08316	3′-3′	598264	598958	33	60.6%	7	100.0%
cis-NAT-4	scaffold12317	3′-3′	65657	66262	26	65.4%	6	83.3%
cis-NAT-5	scaffold03802	3′-3′	435292	436201	4	100.0%	24	54.2%
cis-NAT-6	scaffold11581	3′-3′	786965	787920	7	85.7%	15	53.3%
cis-NAT-7	scaffold06688	3′-3′	321683	322092	8	87.5%	7	85.7%
***Castor bean***								
cis-NAT-8	29701	5′-5′	185445	186292	112	94.6%	152	94.7%

^a^OL stands for overlapping regions.

^b^Abundance of 21-nt reads expressed as the percentage of the total reads.

Unlike miRNAs, many of which are highly conserved in plants [15], the majority plant cis-nat-siRNAs are not conserved [26]. Interestingly, one cis-NAT pair detected in Cassava (013132 m and 020539 m) was conserved in *Arabidopsis* (AT1G01720/AT1G01725; Additional file 1: Figure S10A), which overlap by ~700-nt in the 3′-3′ orientation based on the *Arabidopsis* gene and EST annotation (red box in Additional file 1: Figure S10A). A few small RNAs, 85% of which were 21-nt long, arise from the cis-NAT region in chilling-stressed *Arabidopsis* shoots [26]. In addition, castor bean genome appeared to have one homologous gene of AT1G01720 but no homologous gene for AT1G01725 that was essential for generating cis-NAT1 (Additional file 1: Figure S10B).

### Perturbed sncRNAs in response to chilling stress and acclimation

**Figure 5:**
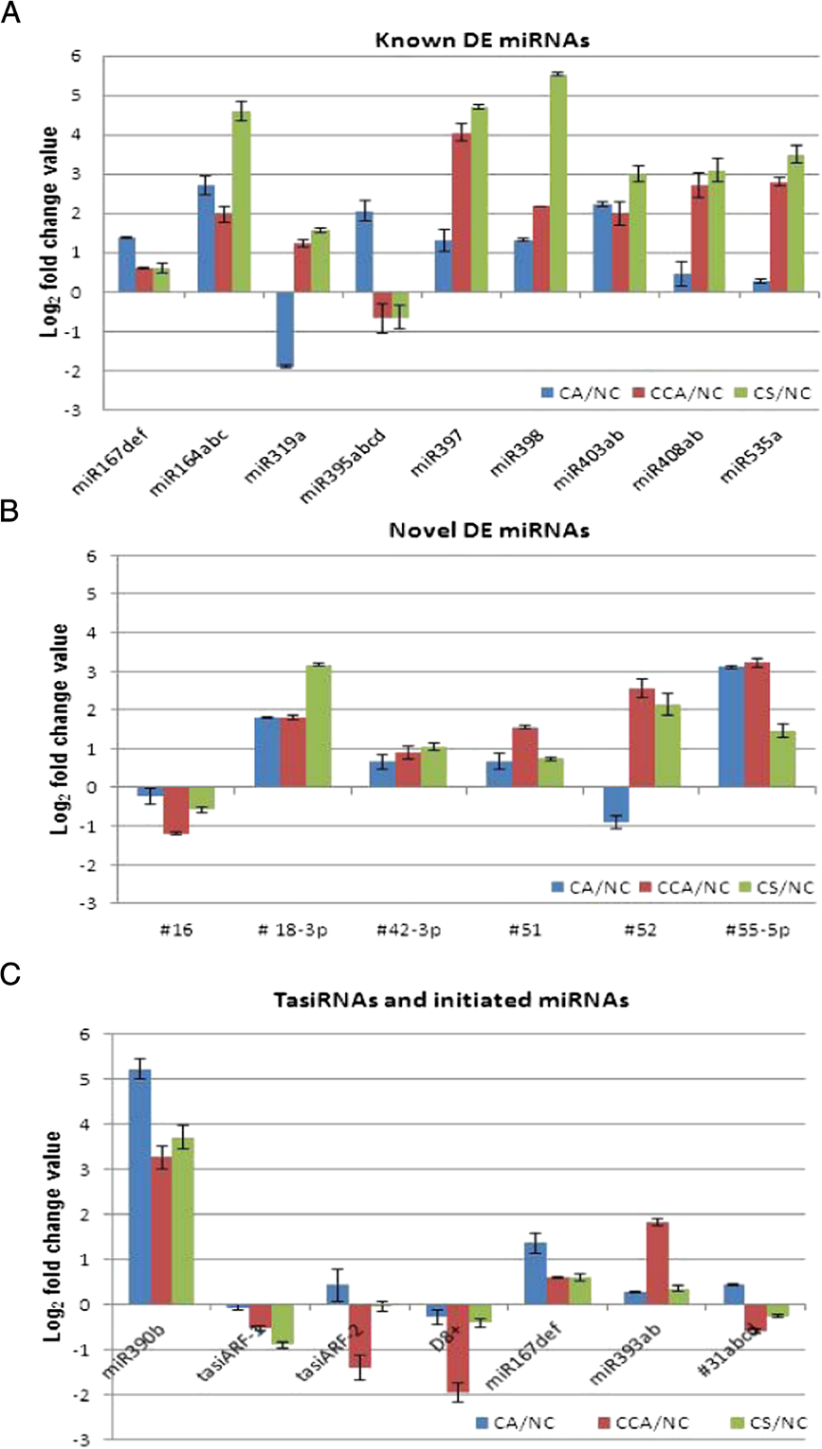
**Experimental validation of differential expression of (A) known miRNAs, (B) novel miRNAs and (C) ta-siRNAs in three chilling stress treatments, CA, CCA and CS, with respect to the normal condition in Cassava SC124 cultivar.**

A substantial number of miRNAs were DE under the chilling stresses with respect to the normal control and were DE across different stress conditions. Among the 150 (111 known and 39 novel and newly detected) miRNAs expressed in Cassava, 114 (78.6% of the total) had at least 1.5-fold change under one of the six comparisons (Additional file 2: Table S12A). Fifteen DE miRNAs were further experimentally analyzed (Figure 5A). Note that the miR319 and miR395 families are well conserved in plants, and miR477 is conserved in Populus, *Arabidopsis*, Grape vine and Moss (Table 2A), suggesting their conserved function in plant stress response. As for the novel chilling-responsive, highly DE Cassava miRNAs, three (novel-51, -52 and novel-55-5p) were up-regulated and one (novel-16) were down-regulated across the chilling treatments. Three novel miRNAs (novel-20, novel-54-5p and novel-55-3p) were not differentially expressed in the three chilling treatments in SC124 cultivar. In contrast, the four miRNAs were all highly up-regulated at 4°C under both CCA and CS of the C4 cultivar (data not shown), a more chilling tolerance cultivar. Consistently, the expression patterns of novel-52 and novel-55-5p were significantly differentially expressed in the three chilling stresses with respect to the control condition, i.e., one was up-regulated and one down-regulated in the CA condition, while both were up-regulated under the CCA and CS conditions (Figure 5B).

The 26 putative siRNA-yielding loci in Cassava were further assessed across six comparisons for their potential for producing differentially expressed siRNAs. In the loci analyzed, 12 (46%) of the 26 loci exhibited differential expression with at least 2-fold change under at least one of the six comparisons (Additional file 2: Table S12B). The miRNAs that initiated siRNAs were highly induced in all three chilling stress conditions compared to NC (Figure 5C). miR167, miR390 and miR393 were also differentially expressed under chilling stress; miR167 was up-regulated under CA while miR393 was up-regulated under CCA in reference to the normal condition. Some of the ta-siRNAs from TAS3 were validated under the four chilling treatments using qRT-PCR. tasiARF-2 and D8+ were down-regulated under the CCA vs NC condition (Figure 5C), indicating that special function of the ta-siRNAs might take part when Cassava was transferred from 14°C to 4°C.

The targets of differentially expressed miRNAs and siRNAs are of special interest, particularly those that have expression patterns that are anti-correlated with that of their regulatory, differentially expressed siRNAs because such relationships provide information of siRNAs’ potential regulatory function. A total of 21 putative targets were recognized to have expression patterns that are anti-correlated with that of differentially expressed targeting siRNAs in the six comparisons considered (Additional file 1: Figure S11). Among the anti-correlated pairs of siRNAs and their targets, many were related to genes involved in chilling-stress and chilling-acclimated response. For example, siRNAs derived from the auxin signaling F-box 3 gene (*AFB3*, 004514 m) was down-regulated in the CA condition with respect to NC and the host gene*AFB3* was also down-regulated in CA vs. NC conditions, as determined by RNA-seq signals. It has been reported that F-Box protein-encoding genes respond to chilling stress in rice [37], leading to changes in gene expression. More importantly, siRNAs from *AFB3* were found to target an amino acid permease gene (005642 m) that was highly up-regulated (>25 fold) in the CA vs NC condition based on RNA-seq profiling. The amino acid permease gene, which functions in amino acid translocation, has been found to be up-regulated in leaves of *Citrus unshiu* when it is fully acclimated after exposure to the chilling. The up-regulated level of the amino acid permease in *C. unshiu* indicates that active transport and metabolism of amino acids are necessary under low temperature conditions, and the roles of the protein has been found to be related to chilling acclimation [38].

While cis-nat-siRNAs from one strand are expected to regulate the gene on the antisense strand of a NAT pair, the differential expression of cis-nat-siRNAs may lead to the anti-correlated expression of their targets [20, 23, 25]. Two of the seven cis-nat-siRNAs were differentially expressed with at least 2-fold changes in at least one of the six comparisons that we considered (Additional file 2: Table S12C). One of these cis-NATs was a gene (013132 m) that was annotated as a NAC domain transcriptional regulator superfamily protein. NAC proteins have been implicated to participate in a wide range of plant developmental processes. Numerous NAC domain proteins have also been implicated in plant abiotic stress responses such as drought and chilling shock response [39].

## Discussion

Plant miRNAs are associated with a variety of biological processes including development and stress responses [4]. Cold inducible miRNAs have been recently studied in variety of plant model species including *A. thaliana*, *O. sativa* (rice) and *T. aestivum* (wheat) [40–43]. The results obtained so far showed that miRNAs play important roles in regulating gene expression in response to chilling stress. The results from the current deep-sequencing based study of two Euphorbia’s species further supported the roles of miRNA in gene regulation upon chilling stress. Not only miRNAs but also tasiARFs were found to be differentially expressed in chilling-stressed condition, indicating a perturbed small RNA system that is potentially associated with the widely altered transcriptome. As a caveat, additional experiments were required to confirm the results from the RNA-seq data.

### Identification of miRNAs and siRNAs in Cassava and castor bean

In addition to an initial set of Cassava miRNAs we identified [30], a substantial number of miRNAs has been reported recently, based on deep-sequencing based profiling or computational prediction [31–33]. Nevertheless, we were able to identify 22 novel miRNAs in Cassava in the current study. Importantly, three novel miRNAs were highly expressed (Table 1A), indicating their important function in Cassava. Furthermore, we used qRT-PCR to validate 4 of the miRNAs that were computationally predicted in previous studies [31] (Additional file 2: Table S4B). We also examined the rest of the candidate miRNAs reported in [31]; however, only a few passed our miRNA identification criteria (see Methods). We thus expect the total number of miRNAs in Cassava to be close to what we report here, whereas the previous study has inflated the number of miRNAs in Cassava.

Here, we identified three TAS3 paralogs in both Cassava and castor bean genomes. While TAS3a is indicated in [13], TAS3b and TAS3c were newly identified in the two genomes in the current study. TAS3c encodes different tasi-ARF siRNAs from TAS3a and TAS3b, and is well conserved in Cassava and castor bean (Figure 2). A careful examination of ta-siRNAs and cis-NATs reported in the previous study [44] showed that the candidates satisfy neither of the two criteria: 1) the enrichment of 21-nt reads within a candidate locus; 2) unique mapping to the candidate locus.

### Differentially expressed sncRNA and auxin response under chilling stress

A recurring theme from our sncRNA profiling and analysis was the set of auxin response genes. For example, miR167 and miR393 were reported to be dysregulated in Cassava of the current study and *A. thaliana* and wheat [40, 42, 43]. More importantly, miR167 has been reported to target auxin response factors, important transcription factors that regulate auxin response genes. Besides, dysregulated miR393 targets auxin signaling F-box, an important auxin receptor gene in the auxin signaling pathway. miR396 has been reported to target growth regulating factors (GRF), known transcription factors that regulate cell proliferation in *Arabidopsis* leaves [45]. Moreover, In *A. thaliana*, miR165 and miR166 have been implicated in regulating a set of targets involved in stress which have enriched functions of vascular development and growth [40, 41]. The common theme of these chilling-responsive miRNAs is the regulation of auxin response and growth regulating genes, which are central players of many important aspects of plant growth and development as well as response to environmental stresses [46–49].

For ta-siRNAs, tasiARFs are well known to target auxin response factors in a wide variety of plant species. tasiARFs are derived from well-conserved TAS3 genes targeted by miR390 in plants. As expected, three TAS3 loci and the targeting miRNA, i.e., miR390, were found to be conserved in Cassava and castor bean. Not only did miRNAs target Auxin response factors, e.g., miR167 targeted ARF8, but secondary tasi-ARFs originating from the three TAS3 genes targeted auxin response factors as well, e.g., tasiARF1 and tasiARF3 targeted ARF3 (002399 m) (Figure 4). We showed that tasiARF1 also targeted auxin response factor 4 (001979 m). Overall, most validated target genes of siRNAs were related to receptors of Auxin signaling, transcription factors and down-stream receptor of Auxin related genes (Table 4). Besides, miR393-mediated auxin signaling F-box (AFB) genes in *Arabidopsis* and our current study on Cassava can further produce siRNAs, which potentially carry out a self regulation on AFB genes [16]. Taken together, both dysregulated miRNAs and siRNAs triggered by miRNAs regulate auxin-related genes, reflecting the robustness and plasticity of small RNA-mediated gene expression regulation.

Auxin is intimately involved in differential growth and bending. For example, root gravitropic curvature is driven by an asymmetric distribution of auxin response pathway [50]. Under a biotic stress, repressing of auxin signaling by miR393 has been shown to contribute to antibacterial resistance to P. syringae in *Arabidopsis* [51]. Under an abitoic stress, it has been shown that chilling stress affects the polar transport of auxin by selective inhibition of intracellular trafficking of auxin efflux carriers [50]. The abnormal auxin among cell, shoot or root and other developmental organs might result in the abnormal development during chilling stress, such as growth retardation, reproduction attenuation and a lack of common survival symptoms observed during chilling attack. Regulation of various factors by miRNAs in the auxin signaling pathway may indicate a conserved role of miRNAs in response to abiotic and biotic stress.

### Cold-responsive miRNAs regulate other important cellular processes in response to chilling stress

In the companion publication [29], we identified a large number of anti-correlated pairs of differentially expressed miRNAs and DE mRNAs. The transcriptome analysis has shown that perturbations of small RNAs as well as variations of transcriptome were most prominent in the CS condition. miRNA-mediated targets were enriched in biosynthetic process, cellular protein modification process, response to stress and metabolic process, consistent with the results from another recent study of Cassava chilling-stressed transcriptome [52]. Particularly, multiple genes in the same enriched processes were regulated by miRNAs under multiple stress conditions. For example, 5 genes (005409 m, 006360 m, 006048 m, 005437 m and 005421 m) targeted by miR399 function in the same biosynthetic process in CA vs. NC. One gene (012052 m) was involved in oxidation-reduction process and 3 genes (033858 m, 014142 m and 000730 m) in metabolic process under CCA vs. NC, which were potentially targeted by miR396a/b/c/d (Additional file 2). Surprisingly, one translation-related gene (018488 m) regulated by miR172 was differentially expressed in four comparisons: CA vs. NC, CS vs. NC, CCA vs. CA and CS vs. CCA. The regulatory roles of miR172 in CA and CS, but not in NC and CCA, may indicate that chilling-acclimated Cassava may reduce energy expense during chilling acclimation and depend on reserved nutrients to combat the adverse effects of chilling stress. In short, miRNAs are indispensable regulating factors for low temperature adaptation in Cassava.

### Secondary siRNAs from disease-related genes

The current study indicated that the majority of secondary siRNA-generating genomic loci correspond to conserved protein-coding genes in Cassava and castor bean. Recent studies have also revealed that a large number of secondary siRNAs, arranged in phasing as ta-siRNAs, arise from disease resistance NBS-LRR-encoding genes initiated by miRNA binding. In this scenario, several master miRNAs regulate a large number of genes in the NBS-LRR gene family. For example, miR1507, miR2118 and miR2109 in *Medicago* target more than 100 NB-LRR-encoding genes to subsequently produce ample siRNAs [18, 19]. Such a siRNA-yielding phenomenon has also been observed in *A. thaliana* and *S. lycopersicum* (tomato) [53–56]. Moreover, different plant species utilize different miRNAs to target genes in the same classes (e.g., NBS-LRR class) for siRNA production. For example, sly-miR482 and sly-miR2118 in tomato and ath-miR472 and ath-miR825 in *Arabidopsis* all target a large number of NBS-LRR genes.

In addition, Cassava miR162 targeted Dicer-like 1 protein and initiated a small amount of siRNAs, which is consistent with the observation in *Arabidopsis* [57]. Remarkably, a large number of miRNA-medicated siRNA-generating loci belong to the coding regions of genes of immune and stress response functions, particularly those disease resistant genes containing the NB-ARC domain. Taken together, a broadly conserved mechanism exists between miRNAs and their target genes in plants; moreover, secondary siRNAs that are derived from the target genes can accumulate to a prominent level, indicating their important function in plants

### Natural-antisense siRNAs

Endo-siRNAs derived from cis-NATs have been widely reported in animal and plants. In *Arabidopsis*, nat-siRNAs from the SRO5 and P5CDH gene pair is induced by salt stress [20]. P5CDH is known as a stress-related gene while the function of SRO5 remains unknown [20]. Other nat-siRNAs from ATGB2 and PPR only accumulates in response to a bacterial pathogen infection. In *Arabidopsis* and rice [22], genome-wide analysis of nat-siRNAs further suggested that the accumulation of many nat-siRNAs is condition-specific [25–27]. Therefore, chilling acclimation and chilling shock were anticipated to induce nat-siRNAs to negate the damage caused by the stress. In the current study, we identified several candidate cis-NAT-siRNA loci which yield a few number of small RNA reads only within the overlapping regions. One of the cis-nat-siRNAs was further found to be differentially expressed under chilling stress compared to the normal condition.

## Conclusion

Our study provided the first results on gene regulation by sncRNAs in chilling acclimation of Euphorbiaceous plants. We identified 83 (61 and 22) novel miRNAs as well as 78 (57 and 21) putative secondary siRNA-yielding and 8 (7 and 1) nat-siRNA-yielding candidate loci in Cassava and castor bean, respectively. We showed above three sncRNA species and mRNA genes in Euphorbiaceous plants experienced dramatic change, especially Auxin response genes, after severe and moderate chilling stresses. This work laid a foundation to elucidate further function of those sncRNA-mediated pathways during chilling stress and acclimation in Cassava.

## Methods

### Plant materials and stress treatments

Stem segments with three nodes of Cassava (*Manihot esculenta* Crantz) cultivar, were cut from 8-month-old plants, and inclined in 3-L pots filled with barren red soil: vermiculite (1:1, v/v), fertilized with Hoagland’s solution [58], to propagate and generate well-balanced seedlings. The solution was renewed with 300 ml quarter-strength solution once a week. After 2 months of planting, the uniform seedlings were subjected to chilling stress treatment. All plants were field grown at the Institute of Tropical Bioscience and Biotechnology (ITBB), Chinese Academy of Tropical Agricultural Sciences (CATAS), Haikou, during April and June of natural conditions (11 h light, 13 h dark and 25°C during the day and 18°C at night).

Two Cassava cultivars (SC124 and C4) were transferred to normal 24°C illumination incubator (SANYO, Japan) for 2 days to set a homogenization starting point, and then subjected to three types of chilling treatment. 1) Gradual *chilling acclimation* (CA in Figure 1): Temperature was decreased from 24°C to 14°C with the rate of -2°C/h to induce mild chilling stress. Temperature was then held constant at 14°C for five days to accommodate chilling acclimation. RNA was collected at 6 h, 24 h and 5d after the temperature reaching14°C. 2) *Cold* stress after *chilling acclimation* (CCA): After 5 days of chilling acclimation and growth under 14°C, plants were watered once with Hoagland’s solution, transferred further to 4°C by -2°C/h gradient cooling, and cultivated at constant 4°C for another 5d. 3) *Cold shock* (CS): Plants grown under 24°C were subjected to dramatic temperature decline to 4°C with a rate of -4°C/h to make sure the temperature reached 4°C at the same time as the CCA treatment. In these two latter treatments, RNA was collected at 6 h, 24 h and 5d after temperature reaching 4°C. In parallel, plants grown under the *normal condition* (NC) of 24°C were watered once with Hoagland’s solution every 5 days, and RNA was collected at 0d, 5d and 10d. At the same time, the NC and CCA treatments of castor bean (cultivar Hela) were carried out as for Cassava. The mixture samples of SC124 (details are given below) were subjected to small-RNA and mRNA expression profiling by NextGen deep sequencing.

### RNA isolation, RNA library preparation and NextGen deep sequencing

Three organs/tissues (folded leaf, fully expanded leaf and roots) from three different Cassava cultivar SC124 plants harvested at 6 h, 24 h and 5d for three chilling treatments of CA, CCA and CS, for profiling genes at the stages of initial response, secondary response, and functional adaption to chilling stresses. Total RNA of each sample was isolated individually, and then pooled with an equal amount from each sample into one for profiling. As a result, four mRNA libraries and four small-RNA libraries, corresponding to the conditions of CA, CCA, CS and NC, were constructed. Similarly, two castor bean small-RNA libraries for the CCA and NC conditions were prepared.

The six small-RNA libraries (four Cassava samples from the NC, CA, CCA and CS conditions plus two castor bean samples from the NC and CCA conditions) were subjected to small-RNA deep sequencing using Illumina GAII. Briefly, total RNA was isolated using RNAplant Reagent kit (TIANGEN, Beijing, China). Small RNAs were enriched by poly-ethylene glycol precipitation, separated on 15% denaturing PAGE, and visualized by SYBR-gold staining. Small RNAs of 16- to 28-nt were gel-purified. Purified small RNAs were ligated to a 5′adaptor and a 3′adaptor sequentially, reverse transcription amplified, and sequenced.

The four mRNA libraries were sequenced by RNA-seq by Solexa GAII following Illumina RNA-seq protocol. Briefly, total RNAs were isolated, purified and reverse transcribed, the resulting cDNA products were subsequently digested with NlaIII and the 3′-cDNA fragments captured with the oligo(dT) beads, and then ligated to the Illumina GEX NlaIII Adapter 1. The junction of Illumina adapter 1 and CATG site contained the recognition site of MmeI, which cut 17-nt downstream of the recognition site (CATG) to produce tags. After removing 3′ fragments with magnetic beads precipitation and MmeI digestion, an Illumina GEX adapter 2 was introduced at the end of tags. The resulting adapter-ligated cDNA tags were subjected to 15 cycles of linear PCR amplified, purified and sequenced with the method of sequencing by synthesis (SBS) using the Illumina Genome Analyzer.

### Preprocessing of small RNA sequencing data

Raw sequence reads that contained no 3′ sequencing adaptor, were of low quality, or were shorter than 17-nt were discarded. The adaptor trimming was done by an in-house method that recursively searches for the longest substring of the adaptor appearing within a sequence read. If a raw sequence read did not have a substring of the adaptor longer than 6-nt, it was considered to have no adaptor. The adaptor-trimmed sequences with no ambiguous reads, which were referred to as qualified reads, were then mapped to the Cassava genome using Bowtie [59].

### Identification of novel miRNAs

Methods for novel miRNA identification were described in our previous papers on miRNAs in other plant species [60, 61]. Here, we briefly discuss the key steps. First, qualified reads in all libraries that mapped to known Cassava (or castor bean) miRNAs (miRBase release 20) were excluded from the identification of novel miRNAs, but were used for re-annotation of known miRNAs. We then mapped the remaining reads to the Cassava genome (or castor bean genome) using Bowtie [59] and merged neighboring loci if they shared overlapping reads. The (merged) loci were extended 300-nt on both ends, and a series of segments of 250-nt were extracted in a sliding window fashion starting from the 5′-end. We then examined the folding structures of the segments using RNA-fold [62]. Candidate miRNAs were chosen based on four key criteria including presence of more than 10 reads, hairpin structures, appearance of miRNA* sequences and RNA-RNA duplex structures with ~2-nt 3′ overhangs.

### Identification of secondary siRNAs

We searched for clusters with 21-nt reads enriched in genomic and cDNA sequences as secondary siRNA-yielding candidates. Specifically, qualified reads from all small-RNA libraries that were aligned to miRNA loci were removed first. The remaining reads were then aligned to genomic and cDNA sequences with Bowtie (version 0.12.7) [59] allowing no mismatches. Genome-aligned reads were clustered within a window size of 50 base pairs to form a putative candidate region. Two stringent criteria were applied to those candidate transcripts and regions with mapped reads. First, candidates with less than 10 mapped reads were removed to ensure a sufficient level of expression. Second, the majority (over 70%) of the mapped reads within a candidate transcript were ensured to be 21-nt long. The two criteria aimed to filter out false positive candidates due to random RNA degradation or other types of endogenous small RNAs that do not possess the characteristic of 21-nt enrichment. These criteria were previously adopted in rice and *Arabidopsis* [16, 63].

We then used TargetFinder [64] to identify putative binding sites of a miRNA on mRNA transcripts. A pair of predicted binding sites on a transcript define a region that serves as a template for synthesizing a dsRNA for siRNA production [8–11]. Candidate siRNA-yielding transcripts were extracted with a flanking extension of 100-nt. Every known and novel miRNA was subject to this analysis. We only considered binding sites with a score from TargetFinder of no less than 4 (see [64] for detail of the scoring metric).

### Identification of cis-NAT pairs and cis-nat-siRNAs

We searched for anti-sense pairs of transcripts (aka, cis-NATs) in the Cassava genome [36] and the castor bean genome [65] which overlapped at least 25-nt at the same genomic loci. cis-NATs were further categorized into three groups: convergent (3′-3′ overlap), divergent (5′-5′ overlap), and enclosed where one transcript was entirely encompassed by the other [25]. We set three stringent criteria for identifying cis-NAT siRNAs, 1) the enrichment of 21-nt reads within a candidate locus; 2) presence of reads uniquely mapped to the candidate locus, and 3) more than 10 reads from the locus. The criteria removed false positive that may be degradation products.

### Identification of differentially expressed sncRNAs

Reads that aligned perfectly to the candidate sncRNA-yielding transcripts were used to compute the digital expression levels of the sncRNAs. Reads mapped to multiple genomic loci were attributed to all derivative small RNAs. Read counts in each sample were normalized to adjust for sample variations. Let *T* be the number of qualified reads that aligned to the genome and cDNA sequences in that sample and *C* the average value of *T* of all samples. The normalized number of reads for each sncRNA in each sample is (*N*_*smRNA*_ * *C*/*T*), where *N*_*smRNA*_ is the number of raw sequencing reads of the sncRNA. Differentially expressed miRNAs were those that had at least 1.5-fold change across two conditions compared.

### Identification of siRNA targets

Every siRNA with more than 10 sequencing reads was subject to target prediction against cDNA sequences. The reverse complementary sequence of a siRNA was mapped to the cDNA sequences using Bowtie; those sites with no more than three mismatches were considered as putative targets of the siRNA.

### Identification of differentially expressed mRNA genes

Sequencing reads from RNA-seq were aligned to the cDNA sequences of Cassava or castor bean using Bowtie (version 0.12.7) allowing no more than one mismatch. The number of mapped reads on each transcript was recorded as a raw read count. A gene was considered as expressed if it has at least 10 CPM (Count Per Million) mapped reads. The genes that had a CPM less than 10 were considered as not expressed. We then normalized the raw read counts of expressed genes using the upper-quartile normalization method. Given two conditions to be compared, a gene was considered to be differentially expressed if either one of the following criteria was satisfied: (1) its normalized count changed at least 4 folds, or (2) the gene was not expressed under one condition but was expressed with more than 40 CPM in the other condition.

### Identification of pairs of anti-correlated sncRNAs and targets

A pair of a sncRNA (a miRNA, secondary siRNA or cis-nat-siRNA) and its target was considered as anti-correlated if the sncRNA was up- or down-regulated and the target was, respectively, down- or up-regulated in the two conditions compared. To filter out possible false positive anti-correlated sncRNA and target pairs we chose targets whose expression changed by at least 2 fold.

### Experimental validation and analysis of sncRNA

The RNAplant Reagent kit (TIANGEN, Beijing, China) was used for total RNA isolation. The quantity and quality of extracted total RNAs was detected by 1% agarose gel electrophoresis and spectrophotometer. This same total RNA sample was used in small RNA and mRNA differential expression assays. A multiplexed RT method was applied to assess the differential expression of selected differential miRNAs. The total RNAs were first-strand cDNA synthesized with pool of miRNA-specific RT primers. These RT primers contain unique tag sequences at the 5′-ends and 7- to 10-nt complementary nucleotides with 3′ ends of specific miRNAs. Real-time PCR was then performed with the cDNA templates generated from the multiplexed RT reaction. The PCR reverse primer specifically anneals to the 5′-end of the cDNA templates, and the PCR forward primer specifically anneals to the tag sequence used in the RT primer. The forward and reverse primers were designed following the strategy by [66], which was developed to amplify mature miRNAs. The amplicons included 21- to 25-nt miRNA specific primers and 30-nt adaptors designed for the common reverse primer template, resulting in ~55-nt target length. The sequences of PCR primers are listed in Additional file 2: Table S13.

Three organs/tissues (folded leaf, fully expanded leaf and roots) from three different Cassava cultivar SC124 plants harvested at 6 h, 24 h and 5d for control and three chilling treatments of CA, CCA and CS, as profiling samples. miRNAs and U6 as the reference gene for each sample were amplified in parallel for 3 replicates. The values of the threshold cycle (CT) were calculated using Rotor-Gene 6000 series software 1.7 (Corbett Robotics, Australia). CT values were converted to relative expression by the ΔΔCT method with the following formula: The relative concentration was 2^–ΔΔCT^, where ΔΔCT = (ΔCTsample –ΔCTcontrol), ΔCT = CT(miRNA)-CT (U6) in each sample. If the CT value was greater than that of one with no template control (NTC), the miRNA was considered not expressed.

### Experimental miRNA target validation

RNA ligase-mediated rapid amplification of 5′cDNA ends (RLM-RACE) GeneRacer Kit (Invitrogen, USA) was used to validate miRNA-guided mRNA cleavage, which differed with traditional 5′RACE of full-length cDNA by omitting the 5′ phosphates of truncated mRNA removal and the 5′ cap structure of full-length mRNA removal treatments. Briefly, total RNA was extracted with RNAplant regent (TIANGEN, DP407-01), and PolyA RNA was isolated using polyAtract mRNA isolation system III (Promega, USA) to eliminate contaminated non-mRNA. Ligation with a 5′ RNA adapter and a reverse transcription were performed then after. The resulting cDNA was used as a template for PCR amplification. Two ~100 bp-spaced gene-specific reverse primers (GSP1 and GSP2) for each target were designed based on the downstream sequence of the miRNA target binding site at the target gene sequence, and combined with two GeneRacer 5′ forward primers (included in GeneRacer kit) to specifically nest amplify the 3′ cleavage product of the target mRNA. The amplified PCR products were gel purified, cloned and sequenced (Sangon, China). Gene specific primers that we used are provided in Additional file 2: Table S14.

### Supporting data

The raw sequencing and processed data from this project have been deposited to the GEO database (http://www.ncbi.nlm.nih.gov/geo/) under the accession number GSE52178.

## Electronic supplementary material

Additional file 1: Figure S1: Sketch of chilling stress experiments for Cassava transcriptome and microRNAome profiling. **Figure S2.** Distributions of length and first nucleotide of sequencing reads in four Cassava small RNA libraries: normal control (NC), chilling acclimation (CA), chilling after chilling acclimation (CCA) and chilling shock (CS). **Figure S3.** Distributions of length and first nucleotide of sequencing reads in two castor bean mall RNA libraries: normal control (NC) and chilling after chilling acclimation (CCA). **Figure S4.** miR390-triggered siRNAs from *TAS3b* in Cassava. **Figure S5.** miR390-triggered siRNAs from *TAS3b* in castor bean. The figures should be read as Figure S5. **Figure S6.** miR390-triggered siRNAs from *TAS3c* in castor bean. The figures should be read as Figure S5. **Figure S7.** Alignment of tasiARF sequences derived from the three TAS3 genes, *TAS3a/b/c*, in Cassava and castor bean. **Figure S8.** Novel miRNA, report-31, triggered siRNAs from an unannotated transcript in Cassava. The figures should be read as Figure S5. **Figure S9.** Experimental validation of secondary siRNAs from TAS3c gene. Amplification curve, dissolution curve, and endpoint gel image of amplicon products of original miRNA and secondary siRNA. Solid arrow refers to the target band in gel. **Figure S10.** cis-NAT pair of Cassava4.1 013132 m and 020539 m were found conserved in Arabidopsis genome but missed in castor bean genome. **Figure S11.** Regulatory networks showing the relationship between DE siRNAs and their anti-correlated target mRNAs. The diamonds indicate siRNA and the circles indicate target mRNAs. (DOC 7 MB)

Additional file 2: Table S1: Statistics of raw sequence reads from four small-RNA libraries from Cassava (A, B and C) under chilling stress and normal condition. **Table S2.** Statistics of RNA-seq data (raw reads and reads mapped to the reference genome with zero mismatches), expressed mRNAs and differentially expressed mRNAs from the normal condition (NC) and three chilling stress conditions (CA, CCA and CS). **Table S3.** Statistics of raw sequence reads from two small-RNA libraries from castor bean under chilling stress and normal condition. **Table S4.** A total of 93 individual miRNAs from 22 families in Cassava were identified by an extended analysis of previously detected miRNAs in Cassava. **Table S5.** Re-annotation of known miRNAs in castor bean. **Table S6.** The experimental validation of known, novel, and some miRNA partners under four chilling treatments in Cassava. **Table S7.** Secondary siRNAs derived from Cassava genes initiated by miRNA targeting. **Table S8.** Secondary siRNAs derived from castor bean genes initiated by miRNA targeting. **Table S9.** Cis-NAT pair loci information in Cassava genome. **Table S10.** Cis-NAT pair loci information in castor bean genome. **Table S11.** RNA-seq signals for the pair of cis-NAT genes in the four conditions. **Table S12.** Differentially expressed sncRNAs in Cassava identified in small RNA sequencing libraries. **Table S13.** The sequences of qRT-PCR primers used in this study. **Table S14.** The two gene specific primers (GSP1 and GSP2) used in the detection of cleavage site of miRNA on it corresponding target genes by 5′RACE experiment. (XLS 214 KB)

Additional file 3: **Alignment of reads to novel Cassava miRNAs.** (TXT 163 KB)

Additional file 4: **Alignment of reads to novel castor bean miRNAs.** (TXT 74 KB)

Additional file 5: **Alignment of reads to known Cassava miRNAs.** (TXT 513 KB)

Additional file 6: **Alignment of reads to known castor bean miRNAs.** (TXT 158 KB)
