# Supplementary material for: Endogenous small-noncoding RNAs and their roles in chilling response and stress acclimation in Cassava
Source: BMC Genomics. 2014 Jul 29;15(1):634. doi: 10.1186/1471-2164-15-634 (PMC4124141; doi:10.1186/1471-2164-15-634)
Supplement: Supplementary file 1 — Additional file 1: Figure S1: Sketch of chilling stress experiments for Cassava transcriptome and microRNAome profiling. Figure S2. Distributions of length and first nucleotide of sequencing reads in four Cassava small RNA libraries: normal control (NC), chilling acclimation (CA), chilling after chilling acclimation (CCA) and chilling shock (CS). Figure S3. Distributions of length and first nucleotide of sequencing reads in two castor bean mall RNA libraries: normal control (NC) and chilling after chilling acclimation (CCA). Figure S4. miR390-triggered siRNAs from TAS3b in Cassava. Figure S5. miR390-triggered siRNAs from TAS3b in castor bean. The figures should be read as Figure S5. Figure S6. miR390-triggered siRNAs from TAS3c in castor bean. The figures should be read as Figure S5. Figure S7. Alignment of tasiARF sequences derived from the three TAS3 genes, TAS3a/b/c, in Cassava and castor bean. Figure S8. Novel miRNA, report-31, triggered siRNAs from an unannotated transcript in Cassava. The figures should be read as Figure S5. Figure S9. Experimental validation of secondary siRNAs from TAS3c gene. Amplification curve, dissolution curve, and endpoint gel image of amplicon products of original miRNA and secondary siRNA. Solid arrow refers to the target band in gel. Figure S10. cis-NAT pair of Cassava4.1 013132 m and 020539 m were found conserved in Arabidopsis genome but missed in castor bean genome. Figure S11. Regulatory networks showing the relationship between DE siRNAs and their anti-correlated target mRNAs. The diamonds indicate siRNA and the circles indicate target mRNAs. (DOC 7 MB) [file 12864_2014_6329_MOESM1_ESM.doc]

**Supplemental Figure S1**. Sketch of chilling stress experiments for cassava transcriptome and microRNAome profiling. Plants grown under the normal condition of 25°C (NC, top left panel) were subjected to a temperature decrease of -2°C/h until reaching 14°C and then cultivated for five days, to reach a state of *chilling acclimation* (CA, top right panel), before RNA was extracted for expression profiling. The chilling acclimated plants were transferred further from 14°C to 4°C by -2°C/h and cultivated for another 5 days, to reach the state of *chilling* stressed after *chilling acclimation* (CCA, low right panel). In contrast, plants gown under the normal condition were subjected to a dramatic temperature drop to 4°C with a gradient of -4°C/h, to reach the state of *chilling shock* (CS, lower left panel). Additional images on leaves are inserted to show the effect of chilling stress on the plants.

**
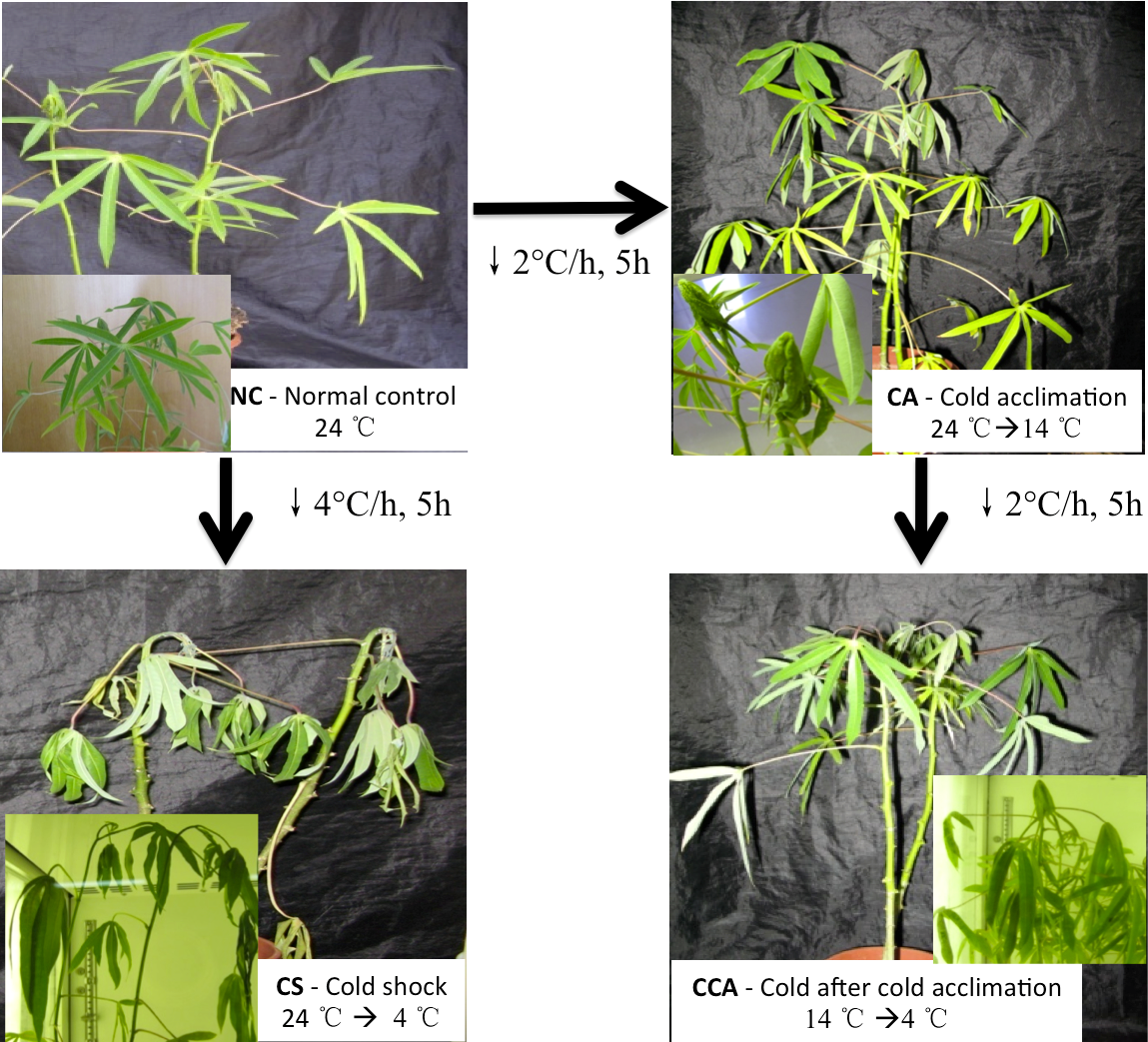
**

**Supplemental Figure S2.** Distributions of length and first nucleotide of sequencing reads in four cassava small RNA libraries: normal control (NC), chilling acclimation (CA), chilling after chilling acclimation (CCA) and chilling shock (CS).

(A) All qualified reads


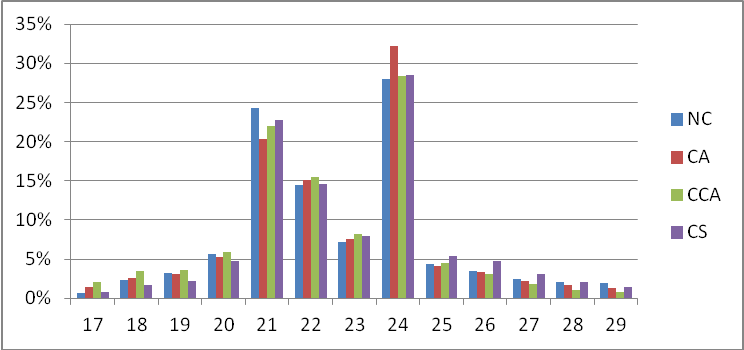

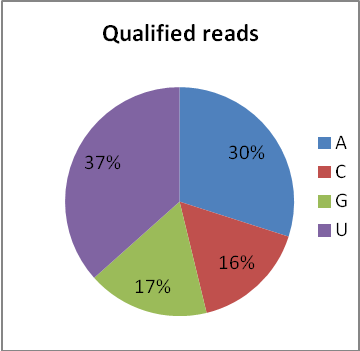


(B) Reads mappable to the genome with one mismatch


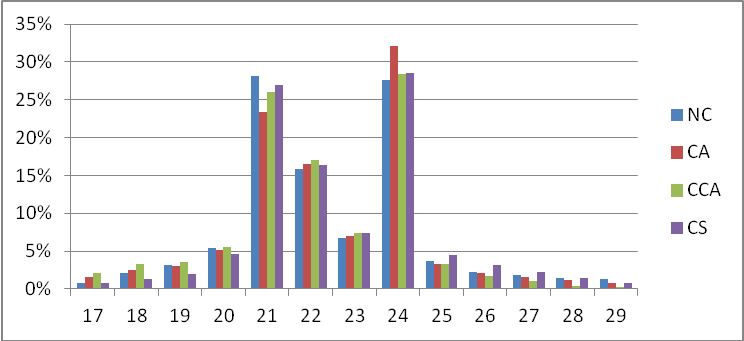

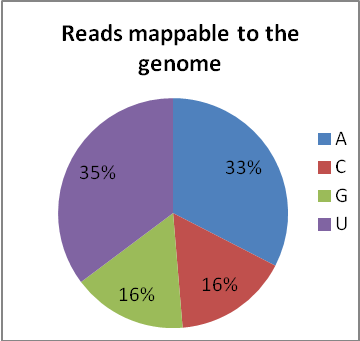


(C) Reads mapped to known miRNAs with one mismatch


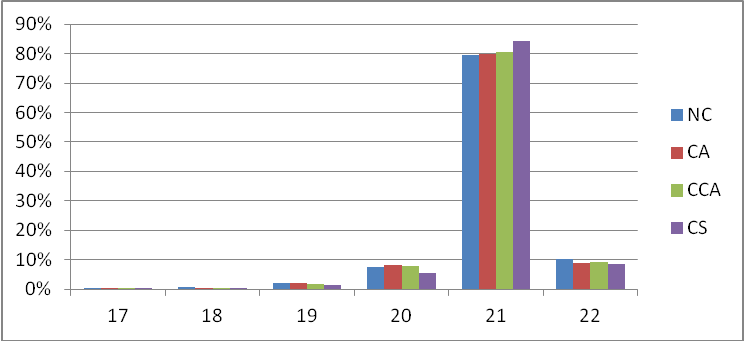

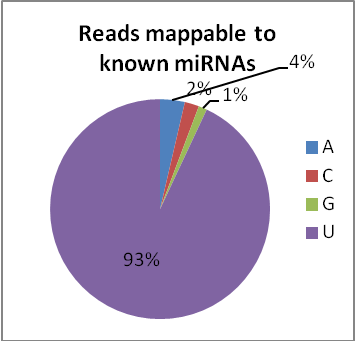


(D) Reads mapped to novel miRNAs with one mismatch


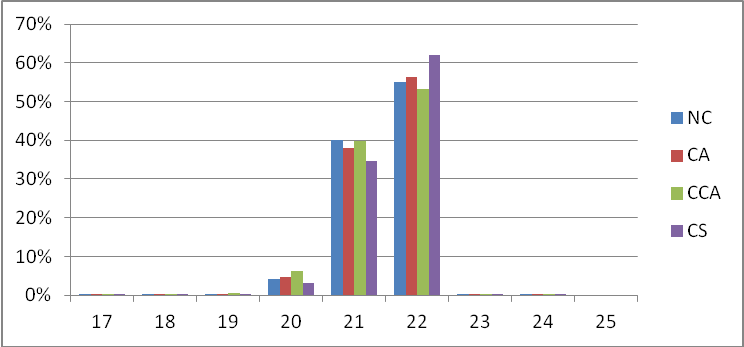

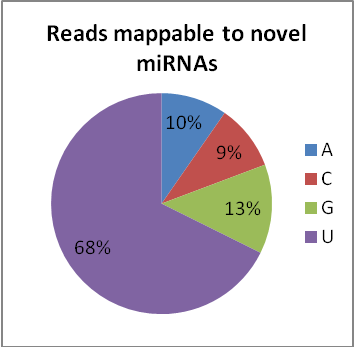


**Supplemental Figure S3.** Distributions of length and first nucleotide of sequencing reads in two castor bean mall RNA libraries: normal control (NC) and chilling after chilling acclimation (CCA).

(A) All qualified reads


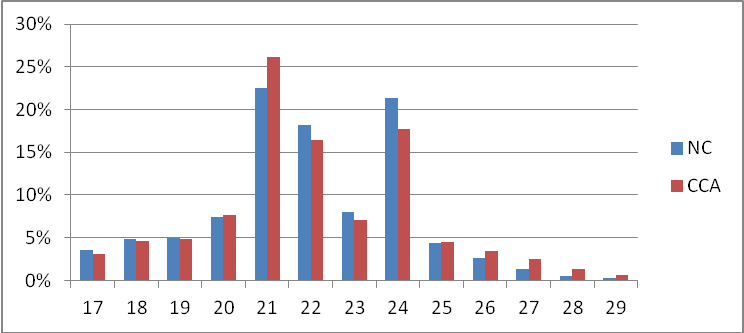

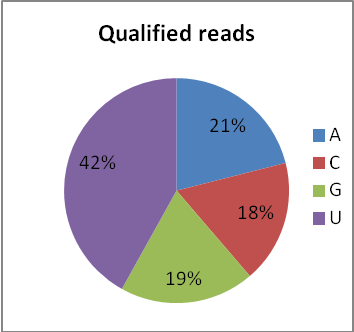


(B) Reads mappable to the genome with no mismatches


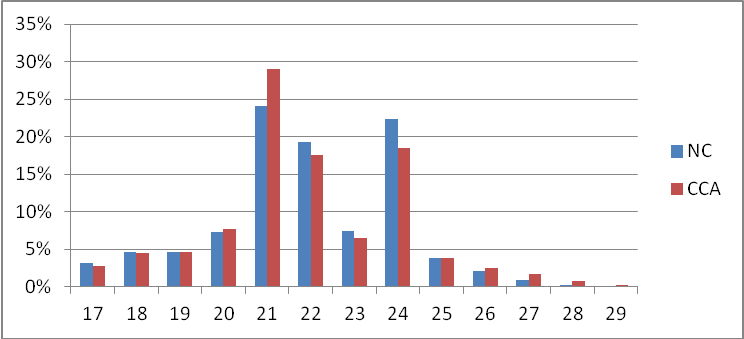

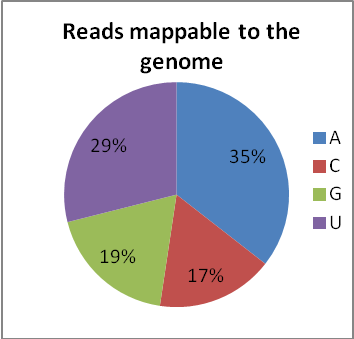


(C) Reads mapped to known miRNAs with no mismatches


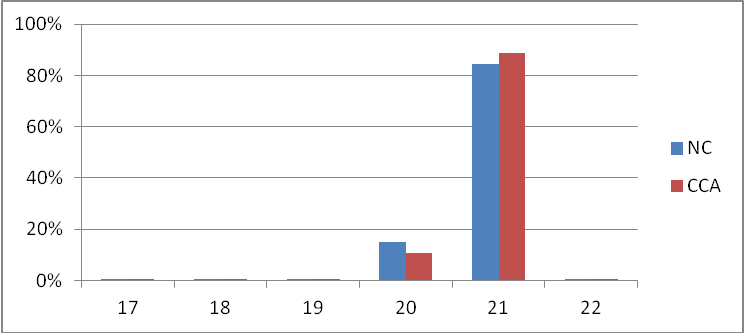

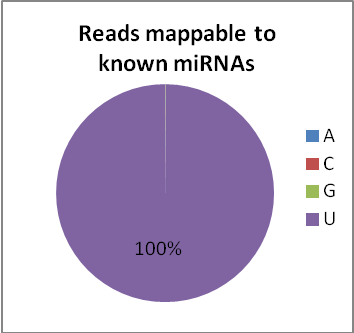


(D) Reads mapped to novel miRNAs with no mismatches


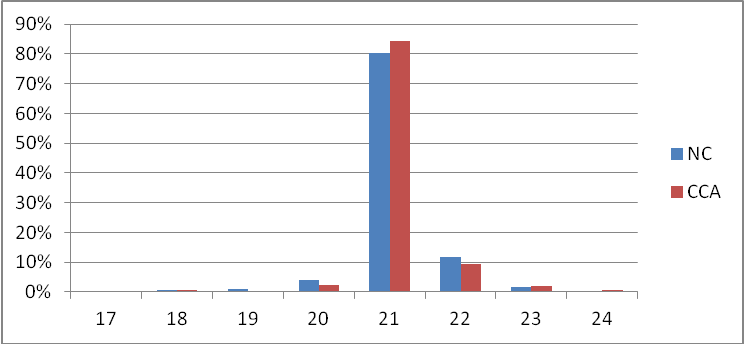

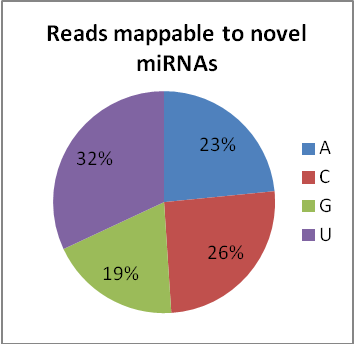


**Supplemental Figure S4**. miR390-triggered siRNAs from *TAS3b* in cassava. (**A**) Distribution of small-RNA reads. Plotted are the 5’ ends of the reads at each position along the sense (in blue) and antisense (in red) strands within the *TAS3b* region. The two arrows marked with miR390 indicate the cleavage sites of miR390 on *TAS3b*. (**B**) The distribution of the lengths of these small RNAs is shown in the right-top figure. (**C**) The radial graph in the right-bottom figure displays the percentage of small RNAs with 5’ ends aligned to one of the 21-phasing registers. The registers to which 5’ and 3’ miR390 cleavage sites are aligned are indicated by miR390 5’ or miR390 3’.


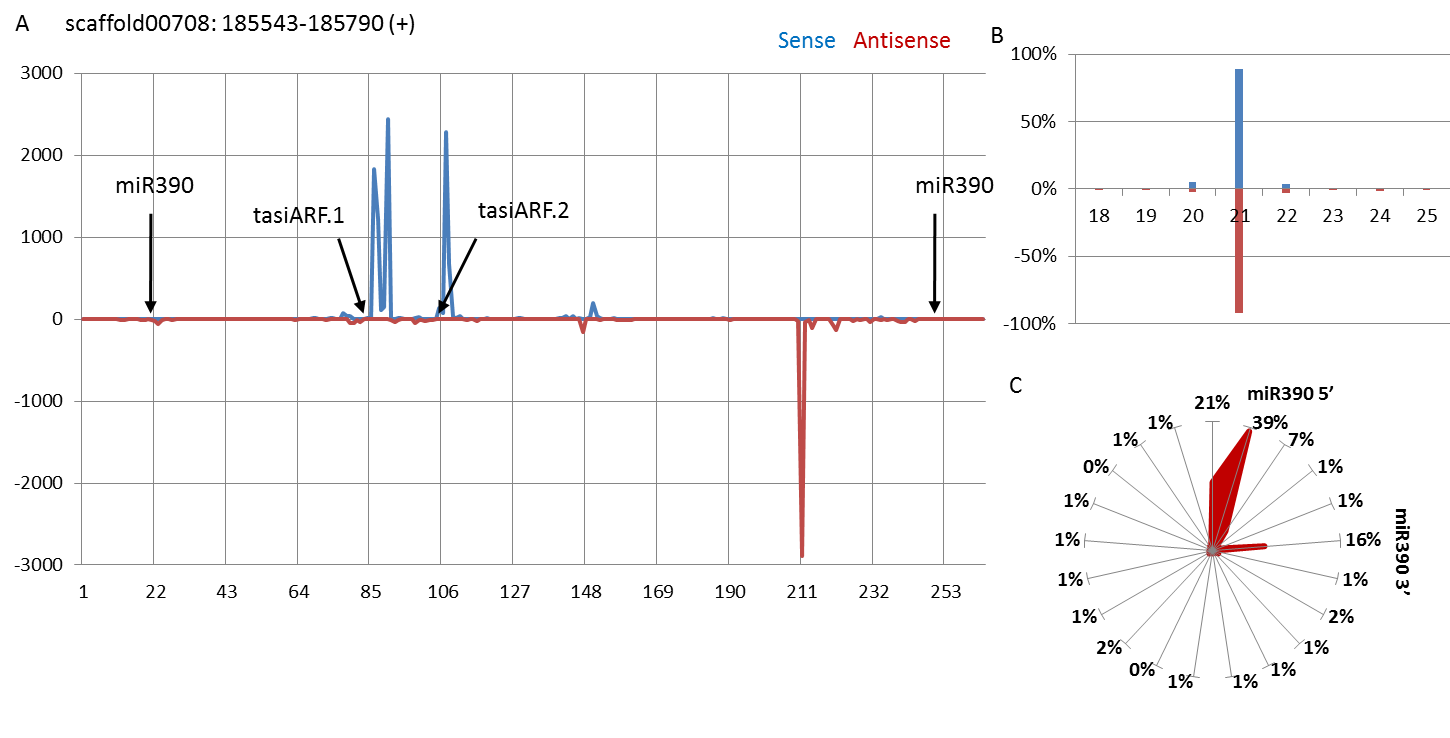


**Supplemental Figure S5**. miR390-triggered siRNAs from *TAS3b* in castor bean. The figures should be read as Figure S5.


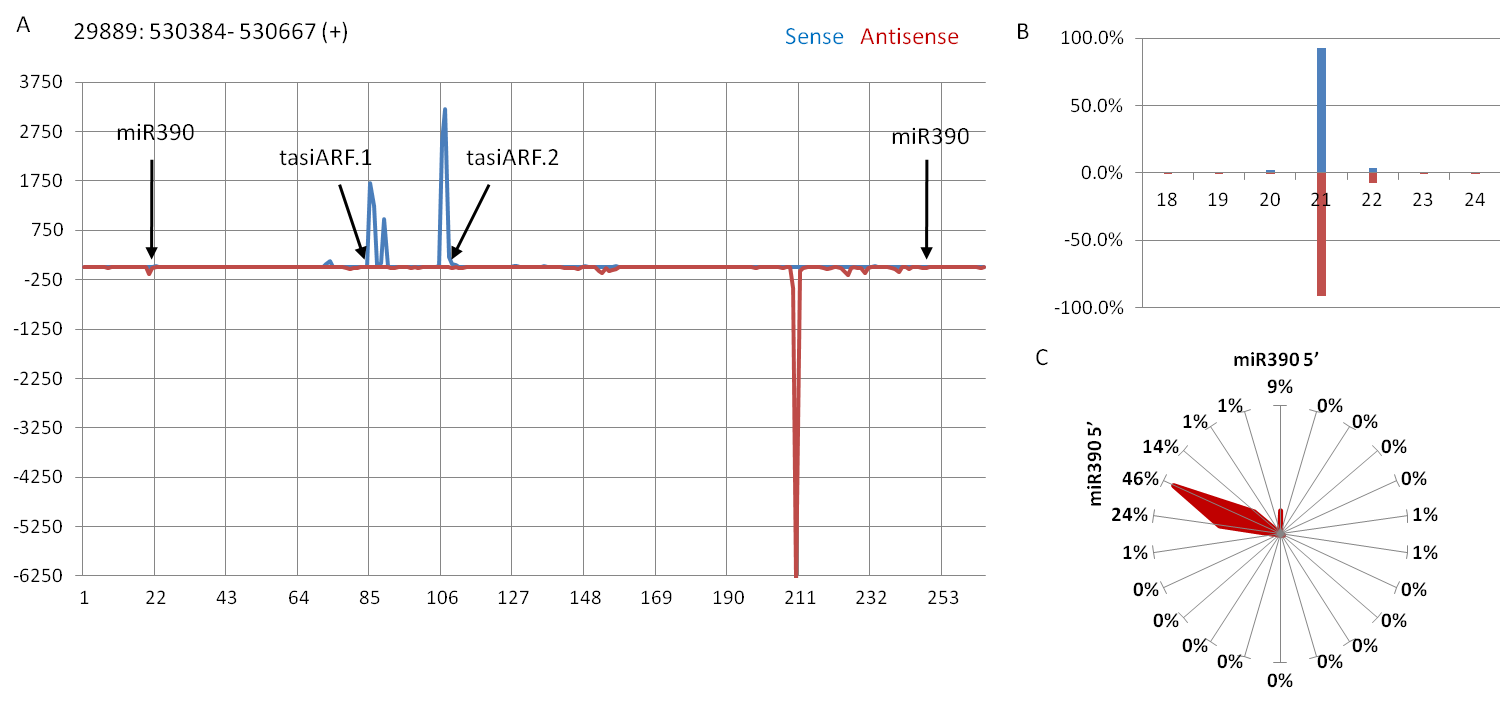


**Supplemental Figure S6**. miR390-triggered siRNAs from *TAS3c* in castor bean. The figures should be read as Figure S5.


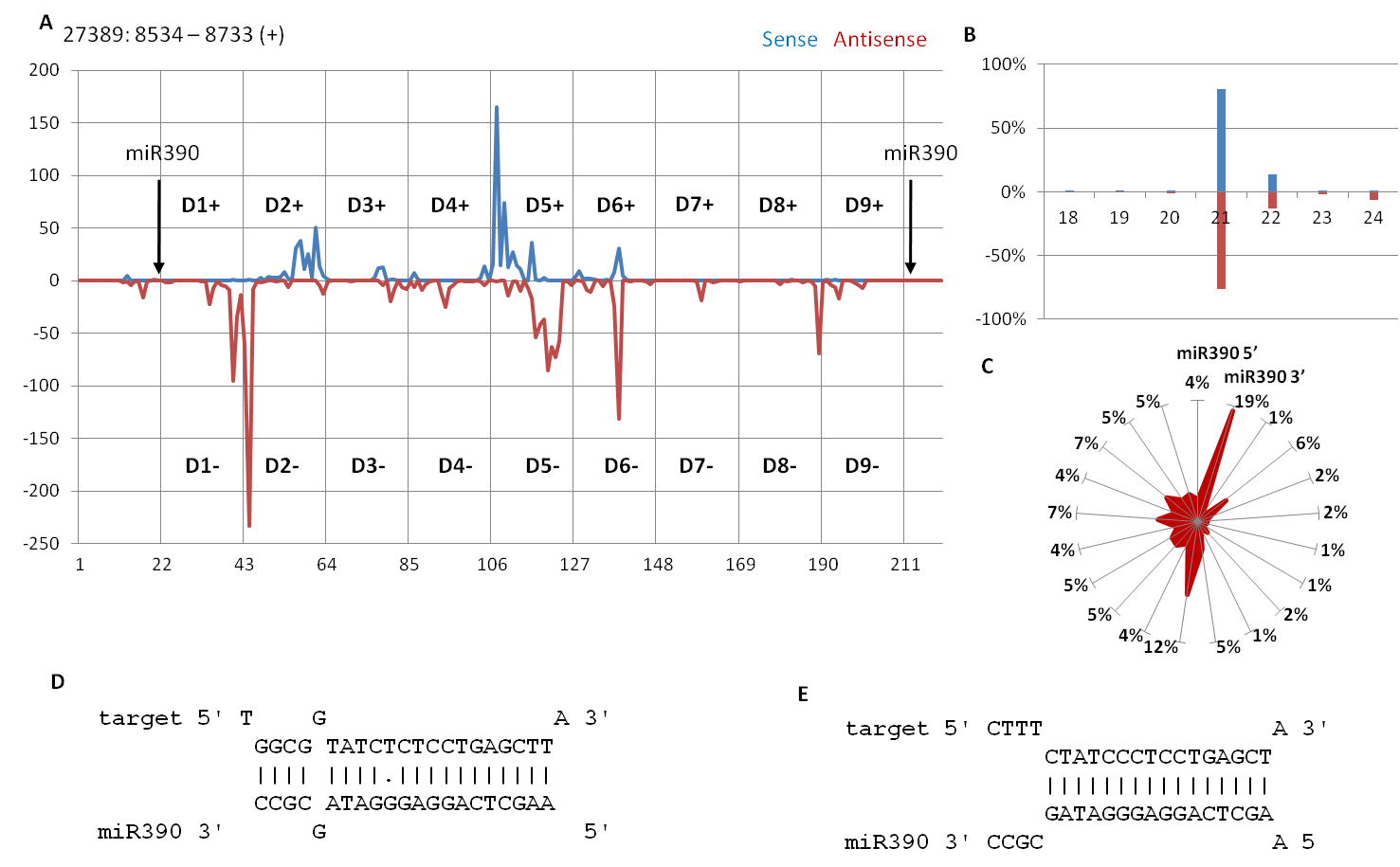


**Supplemental Figure S7**. Alignment of tasiARF sequences derived from the three TAS3 genes, *TAS3a/b/c*, in cassava and castor bean.


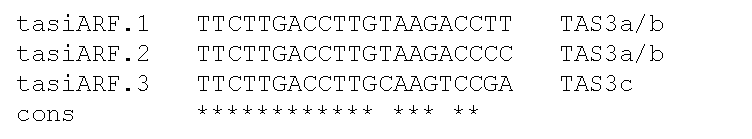


**Supplemental Figure S8**. A recently reported miRNA, reprot-31, triggered siRNAs from an unannotated transcript in cassava. The figures should be read as Figure S5.

**Supplemental Figure S9.** Experimental validation of secondary siRNAs from TAS3c gene. Amplification curve, dissolution curve, and endpoint gel image of amplicon products of original miRNA and secondary siRNA. Solid arrow refers to the target band in gel.


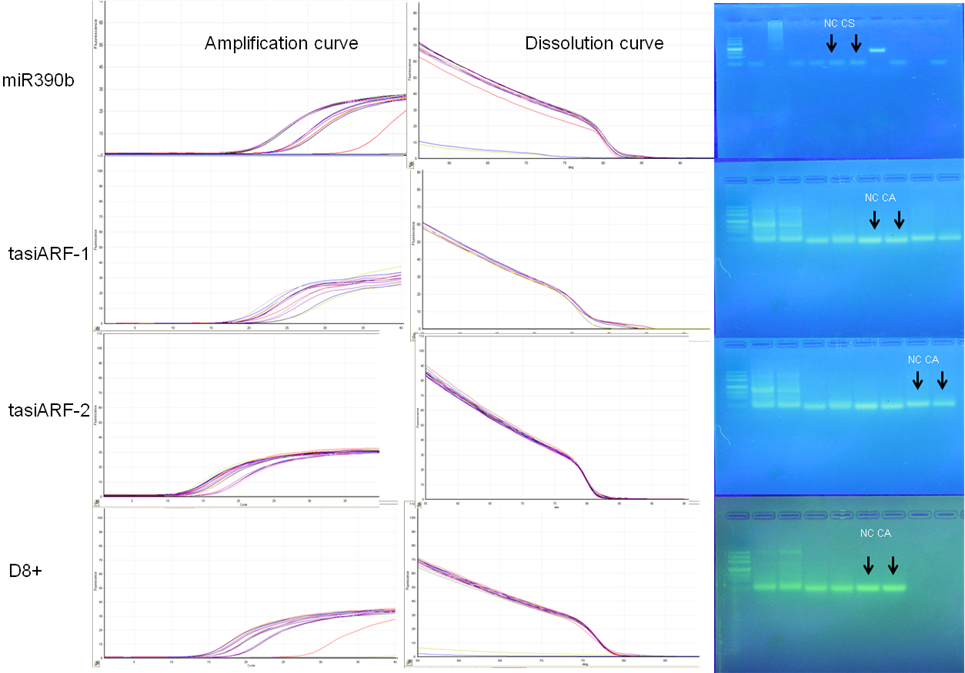


**Supplemental Figure S10**. cis-NAT pair of cassava4.1 013132m and 020539m were found conserved in Arabidopsis genome but missed in castor bean genome.

1. Conserved cis-NAT pair of AT1G01720/AT1G01725 in Arabidopsis genome. The red box shows the overlapping region of the two genes based on Expressed Sequence Tags (EST) sequences from Phytozome database.


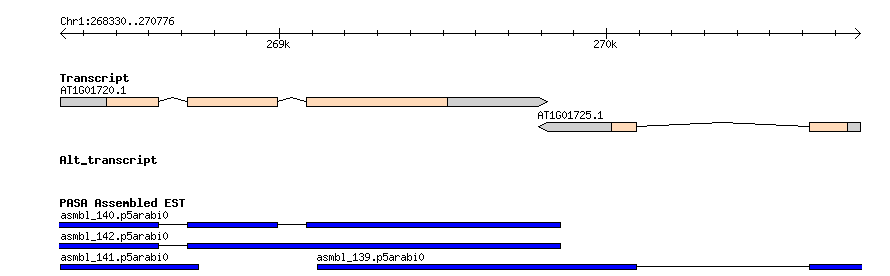


1. The cis-NAT pair was found not conserved in castor bean genome, while only one of the cis-NAT pair, 29648.t000099, is a gene homologous to cassava4.1_013132 in cassava and AT1G01720 in Arabidopsis, while the other gene was not found in castor bean genome.


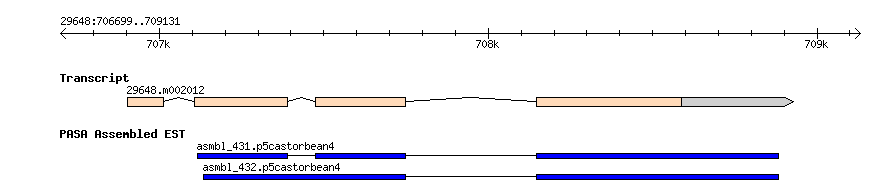


**Figure S11.** Regulatory networks showing the relationship between DE ta-siRNAs and their anti-correlated target mRNAs. The diamonds indicate ta-siiRNA and the circles indicate target mRNAs. An edge between a pair of miRNA and mRNA indicates their anti-correlation relationship three comparisons (**A**) CA vs. NC (**B**) CCA vs. NC (**C**) CS vs. NC.

**
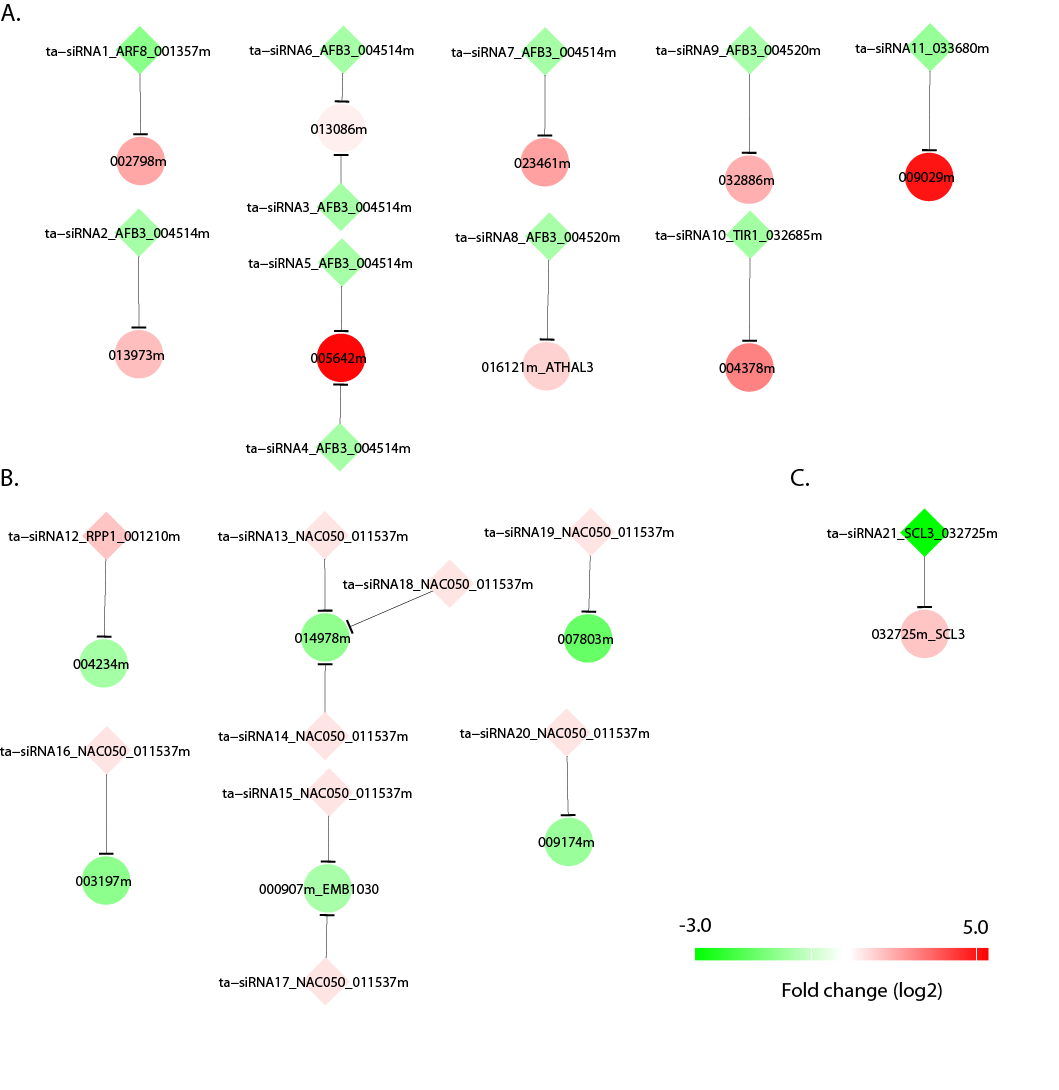
**
